# Genetic architecture and genomic predictive ability of apple quantitative traits across environments

**DOI:** 10.1093/hr/uhac028

**Published:** 2022-02-19

**Authors:** Michaela Jung, Beat Keller, Morgane Roth, Maria José Aranzana, Annemarie Auwerkerken, Walter Guerra, Mehdi Al-Rifaï, Mariusz Lewandowski, Nadia Sanin, Marijn Rymenants, Frédérique Didelot, Christian Dujak, Carolina Font i Forcada, Andrea Knauf, François Laurens, Bruno Studer, Hélène Muranty, Andrea Patocchi

**Affiliations:** Agroscope, Breeding Research Group, 8820 Wädenswil, Switzerland; Molecular Plant Breeding, Institute of Agricultural Sciences, ETH Zurich, 8092 Zurich, Switzerland; Agroscope, Breeding Research Group, 8820 Wädenswil, Switzerland; Molecular Plant Breeding, Institute of Agricultural Sciences, ETH Zurich, 8092 Zurich, Switzerland; Agroscope, Breeding Research Group, 8820 Wädenswil, Switzerland; GAFL, INRAE, 84140 Montfavet, France; IRTA (Institut de Recerca i Tecnologia Agroalimentàries), 08140 Caldes de Montbui, Barcelona, Spain; Centre for Research in Agricultural Genomics (CRAG) CSIC-IRTA-UAB-UB, Campus UAB, 08193 Bellaterra, Barcelona, Spain; Better3fruit N.V., 3202 Rillaar, Belgium; Research Centre Laimburg, 39040 Auer, Italy; Univ Angers, Institut Agro, INRAE, IRHS, SFR QuaSaV, F-49000 Angers, France; The National Institute of Horticultural Research, Konstytucji 3 Maja 1/3, 96-100 Skierniewice, Poland; Research Centre Laimburg, 39040 Auer, Italy; Better3fruit N.V., 3202 Rillaar, Belgium; Laboratory for Plant Genetics and Crop Improvement, KU Leuven, B-3001 Leuven, Belgium; Unité expérimentale Horticole, INRAE, F-49000 Angers, France; Centre for Research in Agricultural Genomics (CRAG) CSIC-IRTA-UAB-UB, Campus UAB, 08193 Bellaterra, Barcelona, Spain; IRTA (Institut de Recerca i Tecnologia Agroalimentàries), 08140 Caldes de Montbui, Barcelona, Spain; Agroscope, Breeding Research Group, 8820 Wädenswil, Switzerland; Molecular Plant Breeding, Institute of Agricultural Sciences, ETH Zurich, 8092 Zurich, Switzerland; Univ Angers, Institut Agro, INRAE, IRHS, SFR QuaSaV, F-49000 Angers, France; Molecular Plant Breeding, Institute of Agricultural Sciences, ETH Zurich, 8092 Zurich, Switzerland; Univ Angers, Institut Agro, INRAE, IRHS, SFR QuaSaV, F-49000 Angers, France; Agroscope, Breeding Research Group, 8820 Wädenswil, Switzerland

## Abstract

Implementation of genomic tools is desirable to increase the efficiency of apple breeding. Recently, the multi-environment apple reference population (apple REFPOP) proved useful for rediscovering loci, estimating genomic predictive ability, and studying genotype by environment interactions (G × E). So far, only two phenological traits were investigated using the apple REFPOP, although the population may be valuable when dissecting genetic architecture and reporting predictive abilities for additional key traits in apple breeding. Here we show contrasting genetic architecture and genomic predictive abilities for 30 quantitative traits across up to six European locations using the apple REFPOP. A total of 59 stable and 277 location-specific associations were found using GWAS, 69.2% of which are novel when compared with 41 reviewed publications. Average genomic predictive abilities of 0.18–0.88 were estimated using main-effect univariate, main-effect multivariate, multi-environment univariate, and multi-environment multivariate models. The G × E accounted for up to 24% of the phenotypic variability. This most comprehensive genomic study in apple in terms of trait-environment combinations provided knowledge of trait biology and prediction models that can be readily applied for marker-assisted or genomic selection, thus facilitating increased breeding efficiency.

## Introduction

Apple (*Malus* × *domestica* Borkh.) is the third most produced fruit crop worldwide [1]. Since its domestication in the Tian Shan mountains of Central Asia, the cultivated apple developed into a separated near-panmictic species [2]. Over the centuries, thousands of apple cultivars have been propagated and conserved thanks to grafting [3]. Extensive relatedness among cultivars with a strong influence of a few founders through the history of apple breeding has been reported despite their high genetic diversity [4–6]. Only a fraction of the existing cultivars are grown commercialy [3] and they require an intensive use of pesticides for crop protection. To diversify apple production, it is desirable to produce new cultivars for sustainable intensive agriculture and adapted to future climates, while remaining attractive to consumers.

Apple breeding is labor- and time-intensive, but selection efficiency can be improved by integrating DNA-informed techniques into the breeding process [7]. Marker-assisted selection allows breeders to predict the value of a target trait based on its association with a genetic marker. The method leads to removal of inferior seedlings without phenotyping, thus increasing selection intensity and/or reducing the labor costs when decreasing the number of individuals passing to the next selection step [7]. Quantitative trait locus (QTL) mapping has been traditionally used to investigate the genetic basis of variation in traits such as pathogen resistance, phenology, and some fruit quality traits [8–11]. To bridge the gap between the discovery of marker-trait associations and their application in breeding, protocols that transfer the knowledge obtained by QTL analyses into DNA tests were established [12, 13]. However, marker-assisted selection in apple remains restricted to a limited number of traits associated with single genes or a handful of large-effect QTL, such as pathogen resistance and fruit firmness, acidity, or color [14]. DNA-informed selection is rarely deployed in apple when breeding for quantitative traits with complex genetic architecture, though this task became feasible with the recent technological developments in apple genomics.

In the genomics era, advancements in genotyping and sequencing technologies led to a broad range of new tools for genetic analyses. In the case of apple, several reference genomes have been produced [15–19], single nucleotide polymorphism (SNP) genotyping arrays of different densities such as 20 K or 480 K SNPs have been developed [20, 21], and genotyping-by-sequencing methods have been adopted [22, 23]. Genome-wide association study (GWAS) emerged as a method for exploring the genetic basis of quantitative traits [24]. GWAS in apple has been used to identify associations between markers and various traits such as fruit quality and phenology traits [22, 23, 25–29]. The associations found with GWAS can be translated into DNA tests for marker-assisted selection. Besides GWAS, genomic selection was developed to exploit the effects of genome-wide variation at loci of both large and small effects on quantitative traits using a single model [30] and is sometimes called marker-assisted selection on a genome-wide scale [31]. For genomic selection, prediction models are first trained with phenotypic and genomic data of a training population. In a second step, the models predict the performance of breeding material based on the genomic data alone. These genomic estimated breeding values are then used to make selections among the breeding material, thus increasing the breeding efficiency and genetic gain. Several studies have assessed genomic predictive ability for apple quantitative traits related to fruit quality and phenology [22, 23, 29, 32–36]. Genomic selection can double genetic gain, as demonstrated by yield traits in dairy cattle [37], but the accuracy of genomic prediction for yield traits in apple has not been studied. Analyses of genomic datasets beyond 100 K SNPs have been limited to flowering and harvest time (GWAS and genomic prediction) [26, 36], fruit firmness and skin color (GWAS) [28, 38]. Marker density, trait architecture, and heritability have been shown to differentially affect prediction performance in simulated data and for apple [34, 36, 39] and their impact on genomic analyses should therefore be further empirically tested. Moreover, GWAS for the same traits measured at different locations, the effect of genotype by environment interaction (G × E) on genomic predictive ability, and predictions with multivariate genomic prediction models have not been evaluated yet in apple.

Plants are known for their strong phenotypic response to environmental factors, a phenomenon regularly tested in plant breeding using multi-environment trials. In general, when statistical models are applied tomeasurements from multi-environment trials, the effect of environment on individuals remains constant at single locations, but the G × E leads to changes in the ranking of genotypes across locations. With an increasing proportion of G × E effect relative to genotypic effect, both heritability of average effect across environments and response to selection decrease [40]. A noticeable effect of contrasting European environments and G × E on two apple phenology traits – floral emergence and harvest date – has been reported, which demands testing the multi-environment modelling approaches in apple [36]. A location-specific GWAS may be used to identify loci with stable effects across environments and loci specific to individual locations [41]. Multi-environment prediction models can account for G × E by explicitly modeling interactions between all available markers and environments [42]. These models can outperform more simple modelling approaches that ignore G × E [42–44]. Additionally, taking advantage of information that traits provide about one another, a multivariate (also called multi-trait) genomic prediction can be applied. This method may be useful in case the assessment of one trait remains costly, but another correlated trait with less expensive measurement is available or can be assessed more easily [45]. The multivariate prediction can also be extended to a multi-environment approach when treating measurements from different environments as distinct traits [46].

A population of 269 diverse apple accessions from across the globe and 265 progeny from 27 parental combinations originating in recent European breeding programs constituted our apple reference population (apple REFPOP) [36]. The apple REFPOP had a high-density genomic dataset of 303 K SNPs and was deemed suitable for the application of genomics-assisted breeding [36]. Combined with extensive phenotypic information, the apple REFPOP provided the groundwork for marker-assisted and genomic selection across contrasting European environments. Hence, 30 traits related to productivity, tree vigor, phenology, and fruit quality were measured in the apple REFPOP during up to three years and at up to six locations with various climatic conditions of Europe (Belgium, France, Italy, Poland, Spain, and Switzerland). GWAS was performed to dissect the genetic architecture of the studied traits, identify associated loci stable across locations and location-specific loci, and to observe signs of selection on loci of large effect. Integrating genetic diversity of the cultivated apple accessions and progeny with phenotypic data measured in multiple environments, the goal was to estimate predictive ability and patterns of G × E for key traits in apple breeding. Compared to previous genomic prediction studies carried out for a smaller number of genotypes and/or environments, this study aimed to obtain improved predictive ability for productivity, tree vigor, phenology, and fruit quality traits using main-effect univariate, main-effect multivariate, multi-environment univariate, and multi-environment multivariate genomic prediction models. Finally, a critical analysis of our results provided recommendations for future implementation of genomic prediction tools in apple breeding.

## Results

### Phenotypic data analysis

**Figure 1:**
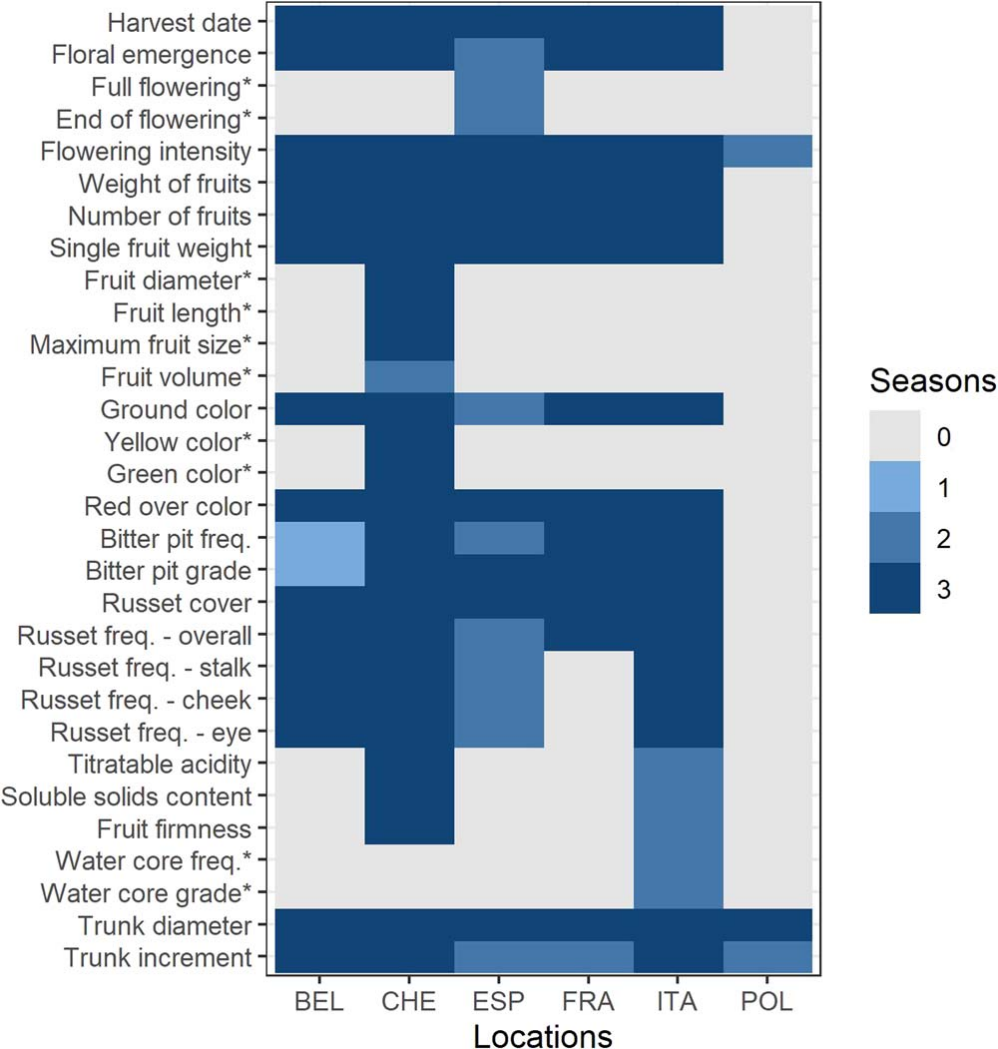
**Locations and the respective number of phenotyping seasons for each trait.** Locations of the measurements are labeled as: BEL – Belgium, CHE – Switzerland, ESP – Spain, FRA – France, ITA – Italy, POL – Poland. Traits measured at a single location are labeled with an asterisk.

**Figure 2:**
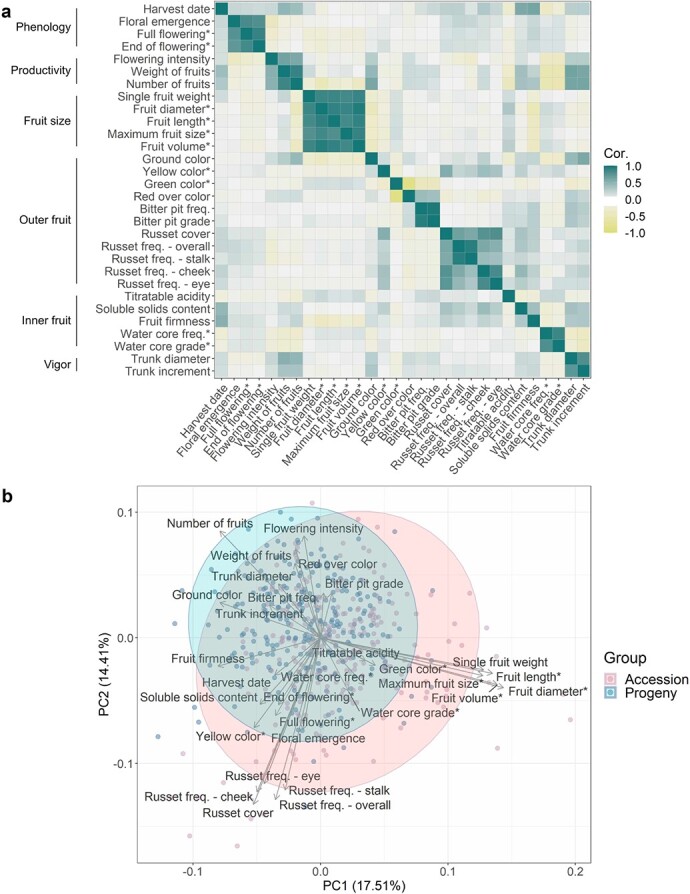
**Exploratory phenotypic data analysis of the studied quantitative apple traits. a** Pairwise correlations between traits with the phenotypic clonal values and genomic breeding values correlations in the lower and upper triangular part, respectively. Phenotypic clonal values correlation was assessed as Pearson correlation between pairs of global clonal values (across-location clonal values with the addition of location-specific clonal values for traits measured at a single location), the genomic breeding values correlation as Pearson correlation between pairs of genomic breeding values estimated from a G-BLUP model. Trait categories are outlined along the vertical axis. Traits measured at a single location are labeled with an asterisk. **b** Principal component analysis biplot based on global clonal values.

The accession and progeny groups of the apple REFPOP were evaluated for 30 quantitative traits at up to six locations. The measurements for ten traits were collected at one location, while the remaining 20 traits were available from at least two locations (three traits were measured in two locations, three traits in four locations, eleven traits in five locations and three traits in six locations, Figure 1, Supplementary Table 1). Most traits [25] were assessed during three seasons while five traits were measured during two seasons. The traits showed unimodal as well as multimodal distributions (Supplementary Figure 1). Differences of various extent between the accession and progeny groups were observed (Supplementary Figure 2). Removing environmental effects from the phenotypic data, best linear unbiased prediction of random effects of genotypes, hereafter called clonal values, were produced across all locations and separately for each location. As expected, high phenotypic clonal values correlations and genomic breeding values correlations (>0.7) between traits were observed within trait categories, namely the phenology, productivity, fruit size, outer fruit, inner fruit, and vigor category (Figure 2a). A few moderate positive phenotypic clonal values correlations (0.3–0.7) were found between trait categories such as harvest date and fruit firmness (0.51), yellow color and russet cover (0.55), soluble solids content and russet cover (0.36), or between yield (weight and number of fruits) and vigor trait category (0.36–0.51, Figure 2a). High average phenotypic clonal values correlations were observed between the environments (combinations of location and year) for harvest date (0.82 [0.73, 0.95]) or red over color (0.80 [0.62, 0.92]) whereas low average phenotypic clonal values correlations (<0.3) were present between environments for flowering intensity (0.18 [−0.49, 0.68]) and trunk increment (0.16 [−0.31, 0.55], Supplementary Table 2, Supplementary Figure 3). A shift of the progeny group compared to the accession group towards smaller, more numerous and less russeted fruits was observed (Figure 2b).

### Genome-wide association studies

**Figure 3:**
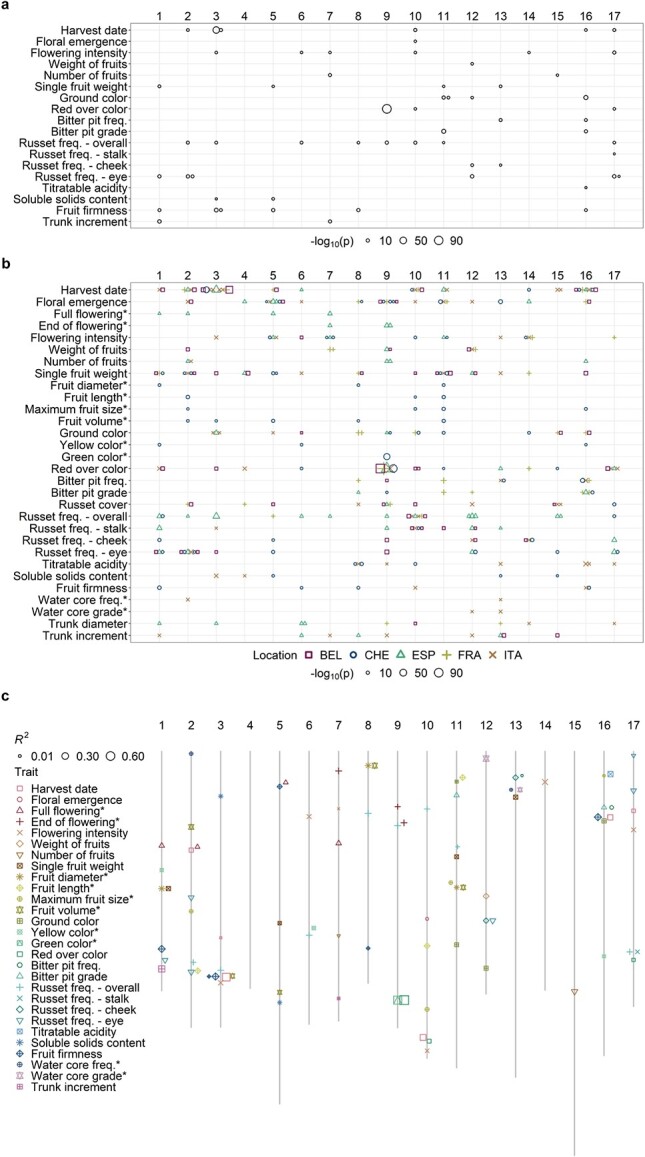
**Significant marker-trait associations found by GWAS. a** Distribution of the significant associations and corresponding p-values from across-location GWAS over the 17 apple chromosomes. **b** Distribution of the significant associations and corresponding p-values from location-specific GWAS over the 17 apple chromosomes. Locations are labeled as BEL (Belgium), CHE (Switzerland), ESP (Spain), FRA (France) and ITA (Italy). **a-b** Size of the symbols indicate the $-{\mathit{\log}}_{10}(p)$. The x-axis shows chromosome numbers. **c** Physical positions (in bp) of the significant associations on chromosomes with their respective coefficients of determination (${R}^2$) from the across-location GWAS complemented with the location-specific GWAS for traits measured at a single location. Size of the symbols indicate the ${R}^2$. The x-axis shows chromosome numbers.

Across-location GWAS for 20 traits measured at more than one location (Figure 1, Supplementary Table 1) and location-specific GWAS for all 30 traits were used to explore the genetic basis of the assessed traits. The quantile-quantile plots showed that the observed and expected distributions of p-values corresponded well and no apparent inflation of p-values was found (Supplementary Figure 4 and 5). Across-location GWAS revealed 59 significant ($-{\mathit{\log}}_{10}(p)>6.74$) marker-trait associations in 18 traits (Figure 3a, Supplementary Table 3). No significant associations were observed for trunk diameter and russet cover in the across-location GWAS. In the location-specific GWAS, 309 significant marker-trait associations for all 30 traits were discovered (Figure 3b, Supplementary Table 3). Of these 309 marker-trait associations, 32 associations for twelve traits were shared between the location-specific GWAS and the across-location GWAS (Supplementary Table 3). The coefficient of determination (${R}^2$) of significant associations was the largest for red over color (0.71), green color (0.55) and harvest date (0.42, Figure 3c, Supplementary Table 3).

Significant associations with different traits co-localized at identical positions or occurred very close in some genomic regions (distance between marker positions below 100 kb, Figure 3c, Supplementary Table 3). In the across-location GWAS, a marker significantly associated with harvest date on chromosome 3 (position 30 681 581 bp) was located next to two markers associated with fruit firmness (positions 30 587 378 and 30 590 166 bp). The same marker on the position 30 681 581 bp was also associated with harvest date, ground color, overall russet frequency and soluble solids content measured at several different locations (location-specific GWAS). Similarly, the association with harvest date on chromosome 16 (position 9 023 861 bp) was closely located to a marker associated with fruit firmness (position 8 985 888 bp) in the across-location GWAS. The traits related to bitter pit analyzed in the across-location GWAS, i.e. bitter pit frequency and grade, showed significant associations on chromosome 16, position 7 681 416 bp. Several associations with traits measuring fruit skin russet in the across-location GWAS co-localized on chromosome 12 (position 23 013 281 bp, russet frequency on cheek and in the eye) and 17 (position 27 249 890 bp, overall russet frequency and russet frequency at stalk). A marker at position 18 679 105 bp on chromosome 1 was associated with both single fruit weight from the across-location GWAS and fruit diameter from Switzerland (found with the location-specific GWAS). The association with marker at position 2 005 502 bp on chromosome 8 was shared between fruit diameter and fruit volume from Switzerland and single fruit weight from Belgium. On chromosome 11, fruit diameter, fruit volume and single fruit weight from Switzerland, as well as single fruit weight from Belgium, shared the association at position 18 521 895 bp. Additionally, position 3 622 193 bp on chromosome 11 was shared between the associations of fruit length and single fruit weight from Switzerland. For red over color and green color, the association with a marker on chromosome 9 (position 33 799 120 bp) occurred in across-location and four location-specific GWAS, while a close marker (position 33 801 013 bp, less than 2 kb away) was associated in the two other location-specific GWAS. Additional significant marker-trait associations occurred in the same genomic regions among the location-specific GWAS and between the across-location and location-specific GWAS (Supplementary Table 3).

**Figure 4:**
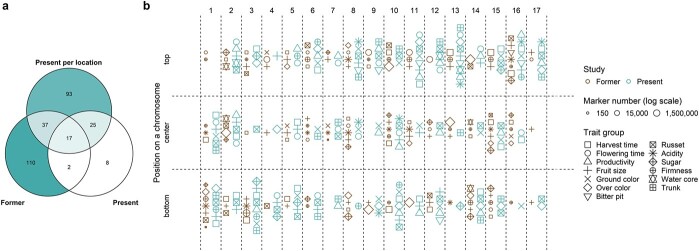
**Comparison of the significant marker-trait associations with previously published associations. a** Venn diagram comparing the unique associations, which were either previously published (former), reported in the across-location GWAS (present) or the location-specific GWAS (present per location). Color intensity and the values reflect the number of associations per diagram area. **b** Scatterplot of unique associations comparing published associations (former) with the merged across-location and location-specific GWAS (present). The traits were assembled into trait groups based on similarity between the approaches to the trait measurement. Symbol size reflects the number of markers used in the studies. In case more than one publication reported an association in the same chromosome segment, only the report with the largest number of markers is shown (see Supplementary Table 4 for the complete list of previously published associations). **a-b** Positions of associations were assigned to three chromosome segments: top, center and bottom. Only the unique combinations of trait groups with segments and type of study (former or present) are shown.

Previous reports on QTL mapping and GWAS in apple were extensively reviewed and 41 publications reporting on traits measured similarly to our own were found and taken for comparison (Supplementary Table 4). In the literature, in the across-location GWAS and in the location-specific GWAS, 166, 52 and 172 unique combinations of chromosome segments with traits were discovered, respectively (Figure 4a). Out of all segment-trait combinations across our GWAS, 30.8% overlapped with the previously published results of QTL mapping or GWAS. All previously published segment-trait combinations for the trait groups bitter pit and trunk were also detected in our study, whereas no overlap between the former and present associations was found for ground color and sugar trait groups (Figure 4b, Supplementary Figure 6).

### Allele frequency dynamics over generations

**Figure 5:**
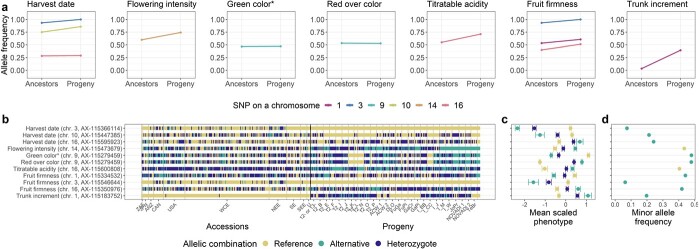
**Allele frequency dynamics of the major significant marker-trait associations. a-d** The associations were chosen based on the coefficient of determination (${R}^2$>0.1) from the global GWAS. **a** For each association, frequency of the allele with increasing effect on trait phenotypes in the apple REFPOP is shown. For the progeny group (progeny) and its five ancestor generations (ancestors), the allele frequencies are shown as points connected with a line. Out of all known ancestors, the allele frequency was estimated for 30 accessions included in the apple REFPOP. Colors of the points and lines correspond to chromosome locations of the associated SNPs. **b** Allelic combinations carried by the apple REFPOP genotypes, sorted according to geographic origin of accessions and affiliation of progeny to parental combinations (the x-axis was labeled according to Supplementary Table 1 and 2 in Jung *et al*. [36]). **c** Global clonal values of traits and their standard error for each allelic combination, centered to mean 0 and scaled to standard deviation of 1. **d** Frequency of the minor allele in the whole apple REFPOP. **b-d** The legend and y-axis are shared between plots. In d, the color of an allele corresponds to the color of the homozygous allelic combination of the same allele in b and c.

Eleven major significant marker-trait associations (${R}^2$>0.1) were identified in the global GWAS results (across-location GWAS with the addition of location-specific GWAS for traits measured at a single location only, Supplementary Table 3). Boxplots of the across-location clonal values against the dosage of the reference allele (0, 1, 2) for the eleven associations showed mostly additive effects of the alleles on phenotypes (Supplementary Figure 7). Among these major associations, changes in the frequency of alleles with an increasing effect on trait phenotypes were quantified in 30 ancestral accessions (five ancestor generations of the progeny group, Supplementary Table 5) and all 265 progenies included in the apple REFPOP (Figure 5a). Compared to the ancestral accessions, the frequency of the allele with an increasing effect on phenotype (Figure 5c) was higher in the progeny for the alleles associated with later harvest date and increased flowering intensity, titratable acidity, fruit firmness and trunk increment (Figure 5a). For the marker associated with green color and red over color, the allele frequencies were equivalent for ancestors and progeny, which reflected the minor allele frequency of nearly 0.5 for both traits (Figure 5b,d).

On a closer look at the allele frequencies across the accession and progeny groups for the markers closely associated with harvest date and fruit firmness on chromosome 3 (Figure 5b), the allele associated with later harvest date and firmer fruits was fixed in all progeny, while the allele with a decreasing effect on the phenotype was present with a frequency below 0.1 in the whole apple REFPOP (Figure 5a-d).

The allele associated with larger trunk increment on chromosome 1 was found in progeny known to segregate for *Rvi6*, and it was present in only two accessions (‘Prima’ and X6398) that are also known to carry the apple scab resistance gene *Rvi6*, which is located about 1.8 Mb from the SNP associated with trunk increment (Figure 5b-c).

Squared Pearson’s correlations in a window of ~3000 markers surrounding each of the major significant marker-trait associations showed that markers in linkage disequilibrium extended over larger distances around some marker-trait associations (Supplementary Figure 8). When visually compared with other loci, the associations with harvest date and fruit firmness on chromosome 3 as well as red over color and green color on chromosome 9 were found in genomic regions of the highest linkage disequilibrium between markers (Supplementary Figure 8). The markers associated with trunk increment and *Rvi6* also showed signs of linkage disequilibrium among them (Supplementary Figure 8).

The remaining associations (${R}^2$≤0.1) reported by the global GWAS showed various trends in allele frequencies across generations such as increased frequency of alleles associated with increased weight of fruits in the progeny (Supplementary Figure 9). The individual parental combinations of the progeny group were often fixed for single alleles of the remaining associations (${R}^2$≤0.1) from the global GWAS (Supplementary Figure 10).

Allele frequencies equivalent to those observed for the whole progeny group were also found for the mean allele frequency for 10-times repeated resampling of 30 genotypes from the progeny group (Supplementary Figure 11).

### Genomic prediction

**Figure 6:**
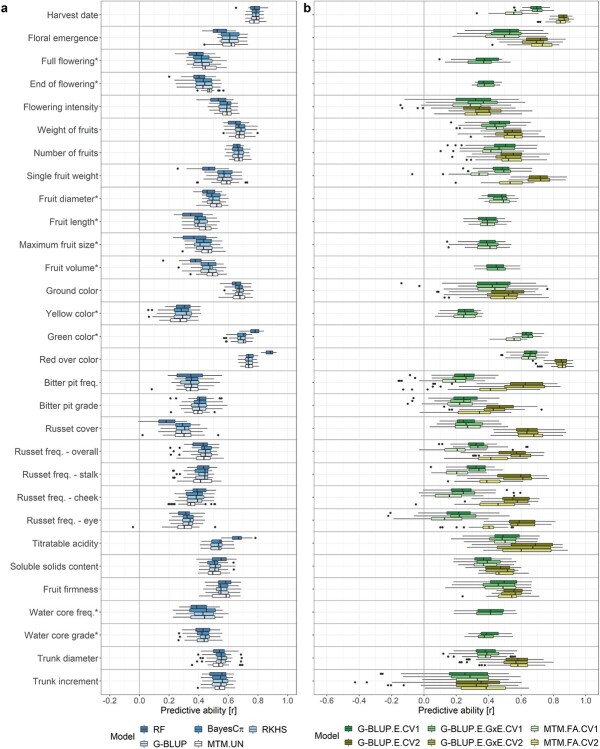
**Genomic predictive ability in apple quantitative traits using eight genomic prediction models and two cross-validation scenarios**. **a** Predictive ability of four main-effect univariate models, i.e. random forest (RF), BayesCπ, Bayesian reproducing kernel Hilbert spaces regression (RKHS) and genomic-BLUP (G-BLUP), and one main-effect multivariate model with an unstructured covariance matrix of the random effect (MTM.UN). The models were applied with a five-fold cross-validation where 20% of the genotypes were masked in each of the five runs. The MTM.UN was used in case a trait showed genomic breeding values correlation larger than 0.3 with at least one other trait. **b** Predictive ability of two multi-environment univariate models, i.e. across-environment G-BLUP (G-BLUP.E) and marker by environment interaction G-BLUP (G-BLUP.E.G × E), and the multi-environment multivariate factor-analytic model (MTM.FA). The models were applied under two five-fold cross-validation scenarios CV1 and CV2. The CV1 was applied for all traits using G-BLUP.E and G-BLUP.E.G × E and for traits measured in at least three environments using MTM.FA. The CV2 was applied for traits measured in Switzerland and in at least a one other location. **a-b** Predictive ability was estimated as a Pearson correlation coefficient between the observed and the predicted values of genotypes whose phenotypes were masked in a five-fold cross-validation. For the multi-environment models, the correlation coefficients were estimated for each environment separately. In the box plot, the bottom and top line of the boxes indicate the 25th percentile and 75th percentile quartiles (the interquartile range), the center line indicates the median (50th percentile). The whiskers extend from the bottom and top line up to 1.5-times the interquartile range. The points beyond the 1.5-times the interquartile range from the bottom and top line are labeled as dots.

The best predictive ability across all eight models compared in this study was found for the traits harvest date, green color and red over color (Figure 6, Supplementary Table 6). The lowest predictive ability was found for traits related to bitter pit and russet as well as yellow color. Additionally, the predictive ability for flowering intensity and trunk increment with the multi-environment models remained strongly below the average predictive abilities per trait (${\overline{r}}_t$) of the corresponding main-effect models (i.e. genomic-BLUP (G-BLUP), random forest (RF), BayesCπ and reproducing kernel Hilbert spaces regression (RKHS)).

Similar performance of five main-effect models was observed (Figure 6a). The ${\overline{r}}_t$ estimated for the baseline model G-BLUP varied between 0.28 and 0.78 (Supplementary Table 6). When the predictive ability of the G-BLUP model was averaged over all traits ($\overline{r}$), the obtained $\overline{r}$ was equal to 0.50. RF showed an ${\overline{r}}_t$ higher than G-BLUP for 9 out of 30 traits and an $\overline{r}$ of 0.49. BayesCπ, RKHS and the main-effect multivariate model with an unstructured covariance matrix of the random effect (MTM.UN) showed an $\overline{r}$ of 0.50, 0.51 and 0.50 and exceeded ${\overline{r}}_t$ of G-BLUP in one, twelve and ten traits, respectively. Among the main-effect univariate genomic prediction models, the ${\overline{r}}_t$ ranged between 0.18 for russet cover and 0.88 for red over color, both extreme values observed with RF (Supplementary Table 6).

When compared with G-BLUP, the main-effect multivariate model MTM.UN showed an improved predictive ability for several traits when they were modelled in combination with a correlated trait (genomic breeding values correlation larger than 0.3, Figure 6a, Supplementary Table 6). The inclusion of floral emergence as correlated trait improved ${\overline{r}}_t$ of full flowering from 0.43 to 0.46 and from 0.43 to 0.47 for end of flowering. Similar response was observed for a combination of weight of fruits with flowering intensity that improved ${\overline{r}}_t$ of flowering intensity from 0.58 to 0.59. Fitting the model using fruit length showed an increased ${\overline{r}}_t$ of single fruit weight (difference in ${\overline{r}}_t$ of 0.01) and using single fruit weight led to an increase in ${\overline{r}}_t$ for fruit diameter, fruit length, maximum fruit size and fruit volume (difference in ${\overline{r}}_t$ of 0.01, 0.03, 0.02 and 0.03, respectively). Using soluble solids content resulted in an increase of ${\overline{r}}_t$ for russet cover (difference in ${\overline{r}}_t$ of 0.01), while using russet frequency at cheek led to an improved ${\overline{r}}_t$ of russet frequency at stalk (difference in predictive ability of 0.01). Predictive abilities for all possible combinations of correlated traits can be found in Supplementary Table 7.

When comparing two multi-environment univariate models – across-environment G-BLUP (G-BLUP.E) and marker by environment interaction G-BLUP (G-BLUP.E.G × E) – and the multi-environment multivariate factor-analytic model (MTM.FA), the prediction performance of G-BLUP.E, G-BLUP.E.G × E and MTM.FA was generally lower under the first cross-validation scenario (CV1) than under the second cross-validation scenario (CV2, Figure 6b, Supplementary Table 6). For all traits, the G-BLUP.E.CV1, G-BLUP.E.G × E.CV1 and MTM.FA.CV1 showed lower ${\overline{r}}_t$ than the main-effect G-BLUP, the $\overline{r}$ being equal to 0.40, 0.40 and 0.36, respectively. The G-BLUP.E.G × E.CV1 performed better than G-BLUP.E.CV1 for 14 out of 30 traits. The G-BLUP.E.CV2 and G-BLUP.E.G × E.CV2 outperformed G-BLUP for 13 out of 20 traits. The G-BLUP.E.CV2 and G-BLUP.E.G × E.CV2 both showed $\overline{r}$ equal to 0.57. The increase in ${\overline{r}}_t$ from G-BLUP to G-BLUP.E.CV2 (0.35) as well as from G-BLUP to G-BLUP.E.G × E.CV2 (0.36) was the most pronounced for russet cover. The performance of G-BLUP.E.CV2 and G-BLUP.E.G × E.CV2 remained below the level of G-BLUP predictions for productivity traits (flowering intensity, weight and number of fruits), ground color, soluble solids content, fruit firmness and trunk increment. The G-BLUP.E.G × E.CV2 performed better than G-BLUP.E.CV2 for 8 out of 20 traits. The $\overline{r}$ of MTM.FA.CV2 was equal to 0.52 and therefore similar to G-BLUP, however, the model outperformed G-BLUP for nine out of 20 predicted traits (Supplementary Table 6). The MTM.FA showed higher predictive ability than both G-BLUP.E and G-BLUP.E.G × E for two traits under CV1 and five traits under CV2 (Supplementary Table 6).

### Synthesis of phenotypic and genomic analyses

**Figure 7:**
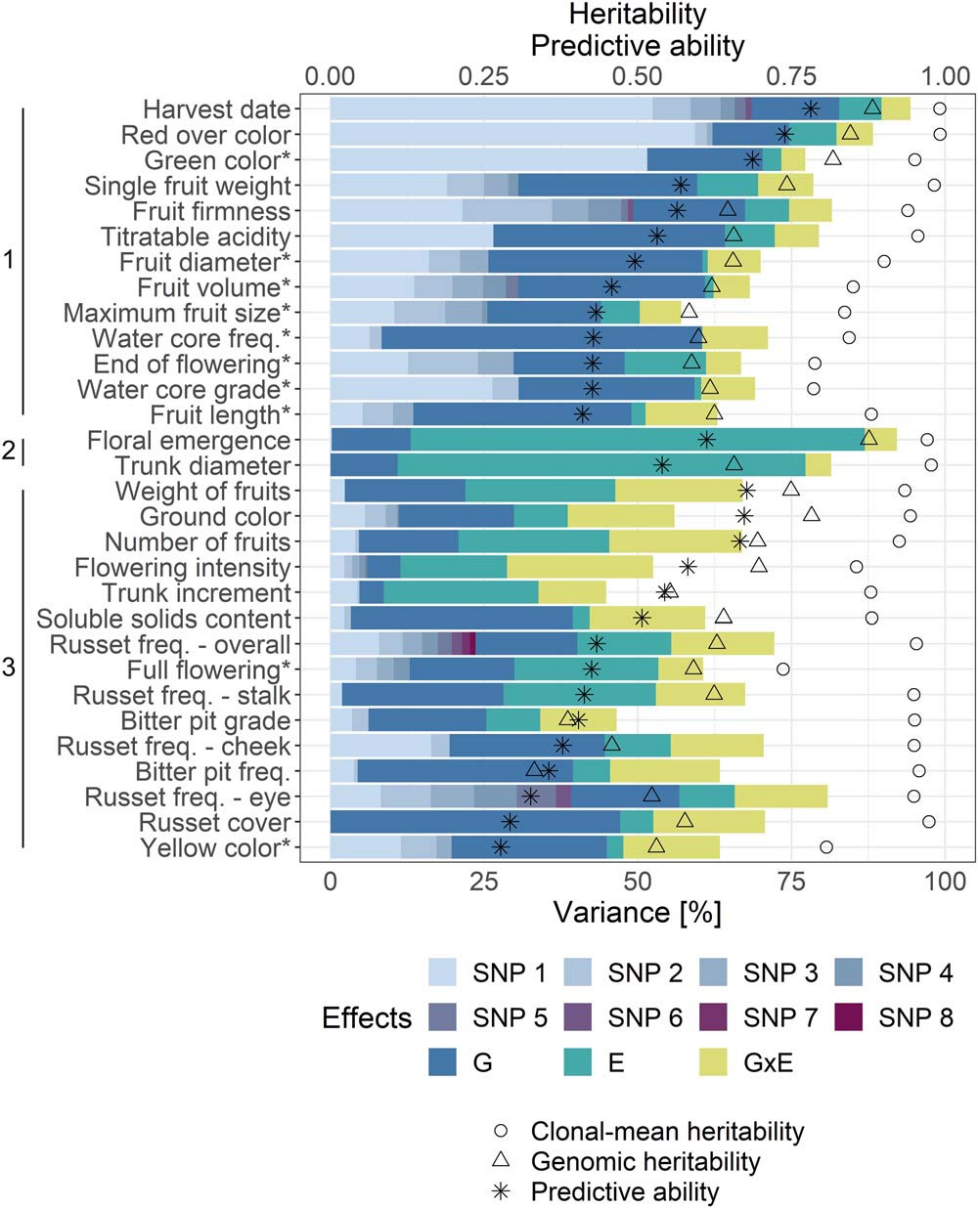
**Synthesis of phenotypic and genomic analyses.** Across-environment clonal mean heritability, genomic heritability, average predictive ability (${\overline{r}}_t$) for the main-effect G-BLUP and the proportion of phenotypic variance explained by the effect of each significantly associated marker (SNP 1–8), genotype (G), environment (E) and genotype by environment interaction (G × E). The significantly associated markers corresponded to results of the global GWAS. SNPs associated with each trait were sorted according to the proportion of phenotypic variance they explained, i.e. SNP 1 represented the association explaining the most variance within a trait. Proportions of phenotypic variance components were used to estimate clusters of traits outlined along the vertical axis. Within each cluster, the traits were sorted according to ${\overline{r}}_t$.

The across-environment clonal mean heritability was generally very high in the evaluated traits, the value being close to one for harvest date and red over color and not lower than 0.80 for all the other traits with the exception of full flowering (0.74), end of flowering (0.79) and water core grade (0.79, Figure 7, Supplementary Table 6). The genomic heritability, which is the proportion of phenotypic variance explained by the markers, was larger than 0.80 for harvest date, floral emergence, green color and red over color, the value was not lower than 0.40 for all the other traits with the exception of bitter bit frequency (0.33) and grade (0.39, Figure 7, Supplementary Table 6).

The effects of genotype and significantly associated markers together explained a substantial part of the phenotypic variance of traits, the largest sums of these genotypic effects were observed for harvest date (82.8%) and red over color (74.6%, Figure 7, Supplementary Table 6). Altogether, the sum of the genotypic effects explained a very low proportion of the total variance for floral emergence (13.1%), flowering intensity (11.4%), trunk diameter (10.9%) and trunk increment (8.7%). The major proportion of the phenotypic variance was explained by the effect of environment for floral emergence (73.9%) and trunk diameter (66.3%). The lowest effect of environment was found for traits measured at only one location over two or three years such as fruit diameter or water core frequency, both showing an effect of environment (i.e. year) below 1%. The effect of G × E was the most pronounced for productivity traits, i.e. flowering intensity (23.7%), weight of fruits (20.8%) and number of fruits (21.6%). The proportion of the G × E effect was the lowest for harvest date (4.7%), floral emergence (5.2%), red over color (5.9%) and trunk diameter (4.2%) among the traits measured at more than one location and for end of flowering (5.7%), fruit volume (5.9%) and green color (3.9%) among the traits measured at one location. A high proportion of the phenotypic variance remained unexplained by the model parameters for flowering intensity (47.5%), bitter pit grade (53.4%) and trunk increment (55.1%).

Three clusters of traits were determined from the hierarchical clustering of the table of traits by proportion of phenotypic variance explained by different sources (Figure 7, Supplementary Figure 12). A strong genotypic effect and a comparably low effect of environment and G × E was observed for 13 traits assigned to the cluster one. Most of the phenotypic variance was explained by the effect of environment in floral emergence and trunk diameter, which were grouped in cluster two. Finally, 15 traits with a pronounced effect of environment and/or G × E were grouped in cluster three.

## Discussion

Both study aims were successfully achieved. Dissection of the genetic architecture of 30 key traits in apple breeding and identification of associated loci stable across locations and location-specific loci using GWAS provided 336 stable and location-specific marker-trait associations discovered across all traits under study. Of these loci, 69.2% were novel when compared with previously published associations. Additionally, signs of selection were found for associations of large effect. The multi-environment and multi-trait experimental design of the apple REFPOP allowed to estimate patterns of G × E for the studied traits and assess their predictive ability. Our results showed that G × E accounted for up to 24% of the phenotypic variability and genomic predictive abilities of up to 0.88 were observed. Based on the outputs of this research, recommendations for future implementation of the prediction tools in apple breeding were derived.

### Discovered loci overlap between association studies and traits

Our GWAS enlightened the architecture of analyzed traits as well as the identification of numerous marker-trait associations stable across, and specific to, the locations of the apple REFPOP. The particular design of the experiment, including the diversity of the plant material used (accessions and small progeny groups), multiple locations, and multiple years of evaluation, are likely to have resulted in about two thirds of the discovered associations being novel when compared with the loci published in studies spanning more than two decades. Our study design also allowed us to replicate the identification of many previously known loci associated with the studied traits.

The association of one locus with two or more seemingly independent traits (i.e. caused by pleiotropy) and linkage disequilibrium between loci associated with different traits are frequent for complex traits [47]. The GWAS performed in this study showed several marker-trait associations at identical or close positions for different traits. The interdependency between harvest date and fruit firmness, which can be also observed empirically for early cultivars that soften more, may be an example of pleiotropy or linkage disequilibrium between loci. Harvest date and fruit firmness are known to be regulated by ethylene production [48] and associated with loci present on chromosomes 3 (*NAC18.1*), 10 (*Md-ACO1*, *Md-PG1*), 15 (*Md-ACS1*) and 16 [22, 49–52].

In this work, closely located (distance <100 kb) associations with both harvest date and fruit firmness were found on chromosome 3. Migicovsky *et al*. [22] reported an overlap between associations with harvest time and fruit firmness on chromosome 3 falling within the coding region of *NAC18.1*. The authors hypothesized that the lack of associations on other chromosomes was likely due to low SNP density around the causal loci (the study used a GBS-derived 8 K SNP dataset for 689 genotypes). The larger number of associations reported here might be a result of the high SNP density (303 K SNPs) deployed in GWAS, however, not all previously reported loci were re-discovered.

The SNPs associated with harvest date and fruit firmness on chromosome 10 were further apart (~6 Mb). For harvest date, one of the associations on chromosome 10 was stable across locations and several associations were location specific. However, the association on chromosome 10 with fruit firmness was found for the Italian location only. It has been shown that chromosome 10 contains more than one QTL controlling fruit firmness [49–51], but stable across-location association with fruit firmness on chromosome 10 was missing in our study. One of the known loci on chromosome 10, the *Md-PG1* gene, is responsible for the loss of fruit firmness after storage [51, 53]. In apple REFPOP, fruit firmness was measured within one week after the harvest date and this very short storage period might have contributed to the less pronounced effect of the locus *Md-PG1* in our GWAS.

Two associations with harvest date measured in Italy but no association with fruit firmness were found on chromosome 15. Although a marker for *Md-ACS1* related to ethylene production was previously mapped on chromosome 15 [50], and QTL for fruit firmness was discovered on the same chromosome [49], these markers did not co-locate, but rather, mapped at the opposite extremes of chromosome 15 [49, 50]. Likewise, the connection between harvest date and fruit firmness on chromosome 15 could not be confirmed here.

Our GWAS showed associations with harvest date and fruit firmness on chromosome 16, which were located 38 kb apart. In the past, loci associated with harvest date and fruit firmness have been reported in the same region on chromosome 16 [26, 49]. The role of this locus in the regulation of harvest date and fruit firmness remains unknown and requires further research.

In practice, ripeness of fruit (harvest date) is decided based on ground color and starch content. The GWAS results showed that the association on chromosome 3 was not only found for harvest date and nearby markers associated with fruit firmness, but also corresponded to associations with ground color and soluble solids content. This might be explained by the fact that these traits are used to define ripeness and thus harvest date. Further, the association of the *NAC18.*1 locus on chromosome 3 with overall russet frequency would support the known enhanced expression of *NAC* transcription factors in russet skin [54].

Co-localizations between associations found for different measures of bitter pit on chromosome 16, russet on chromosomes 12 and 17, fruit size on chromosomes 1, 8 and 11, and skin color on chromosome 9 are likely the result of relatedness among trait measurements. The measures that are easiest to score can be used in future to phenotype these traits.

### Signs of selection in marker-trait associations of large effect

The design of apple REFPOP allowed for the discovery of major marker-trait associations and for the analysis of changes in allele frequency between 30 ancestral accessions and 265 progeny included in the apple REFPOP. Although the progeny group did not undergo phenotypic selection before the establishment of the apple REFPOP, the parents of the progeny were a matter of choice within the European breeding programs. This allowed us to assess the impact of the past phenotypic selection on the studied germplasm. Comparing ancestors with the progeny, higher frequencies of the alleles associated with later harvest date and increased flowering intensity, titratable acidity, fruit firmness and trunk increment were found for the progeny. Of these traits, harvest date and fruit firmness are correlated, probably due to pleiotropy or linkage disequilibrium of causal loci, as it was shown in this and previous studies [22]. Consequently, the consistently higher frequency of alleles contributing to later harvest and firmer apples in the progeny is because the softening of harvested apples is undesirable and likely selected against [55]. Signs of selection for increased firmness were also recently found in USDA germplasm collection [5]. Our study also showed fixation of the late-harvest and high-firmness alleles on chromosome 3 in the whole progeny group, which suggests a loss of genetic diversity in the modern breeding material at this locus. For flowering intensity, a trait positively correlated with apple yield (i.e. weight and number of fruits, Figure 2a), a new locus was discovered on chromosome 14. The increased frequency of the allele contributing to higher flowering intensity in the progeny, its presence in all parental genotypes, and fixation in some parental combinations may be the result of breeding for high yield. The major locus found for acidity on chromosome 16 was consistent with the *Ma* locus frequently detected in various germplasm [8, 11]. The total number of the high-acidity alleles for *Ma* and *Ma3*, which is another regularly detected acidity locus, was shown to be higher in parents of a European breeding program (Better3fruit, Belgium) than in parents used in the US breeding programs [11, 56]. The desired acidity level might depend on local climate of the breeding program and market preferences [56]. The increase in frequency of the allele contributing to higher acidity in the progeny may indicate a current preference towards more acidic apples in European breeding, but further investigation is needed to clarify the trend. The last locus of large effect showing allele frequency dynamics between generations was found for trunk increment. The increased frequency of allele associated with an increase in trunk increment may have occurred in the progeny due to its potential impact on productivity suggested by moderate positive phenotypic clonal values correlations between tree vigor (trunk diameter and increment) and yield-related traits. Additionally, the marker associated with trunk increment was 1.8 Mb apart from a SNP marker associated with *Rvi6* gene responsible for resistance against apple scab [10]. These two markers (AX-115183752 for trunk increment and AX-115182989 (also called Rvi6_42M10SP6_R193) for apple scab) showed a correlation of 0.15 and occurred within a region of increased linkage disequilibrium between markers (Supplementary Figure 8). All accessions were homozygous for the reference allele of AX-115183752 associated with decreased trunk increment (Figure 7c) except for ‘Prima’ and X6398, which were heterozygous. The scab-resistant accessions ‘Prima’ and X6398 (which is a second-generation offspring of ‘Prima’ [57]) but also ‘Priscilla-NL’ (known to be heterozygous for *Rvi6* [58]), were also heterozygous for AX-115182989. All other accessions were homozygous for the reference allele not associated with *Rvi6*. The allele on chromosome 1 associated with increased trunk increment may have been co-selected with the *Rvi6* locus responsible for resistance against apple scab.

Signs of intense selection for red skin were recently detected in the USDA germplasm collection when compared with progenitor species of the cultivated apple [5]. Our results show that the associations with red over color and green color, which phenotypically mirrored red over color and was associated with the same marker, did not show changes in allele frequency between ancestors and progeny included in the apple REFPOP. Some parental combinations showed almost exclusively the allele increasing red skin color, other parental combinations exhibited a lack of the allele. This uneven distribution of the alleles in the progeny group pointed to different directions of selection for fruit skin color in the European breeding programs (Figure 5b).

### Performance of the main-effect univariate genomic prediction models

Main-effect univariate genomic prediction models were applied to the global clonal values. The observed small differences between genomic predictive abilities of various models (Figure 6a) were in accordance with previous model comparisons where distinctions among models were negligible [39, 59]. The extremes in predictive ability between traits were found with random forest, which allowed for the overall highest predictive ability among all compared models for red over color. The explanation for the striking performance of random forest for red over color might be found in the results of our GWAS. This trait of oligogenic architecture was associated with a few small-effect loci and one locus of large effect explaining 61% of the red over color phenotypic variance measured in the apple REFPOP. High correlations between many markers, i.e. linkage disequilibrium, were found in the vicinity of the large-effect locus (Supplementary Figure 8). Random forest is known to assign higher importance to correlated predictor variables (based on the amount of accuracy decrease when a variable – here marker – is excluded during the decision tree construction) [60], which may have contributed to the particularly high predictive ability found for red over color with random forest.

The predictive ability for red over color reached ~0.4 in several former prediction studies [22, 23, 29, 34] and was approximately doubled in our work, which demonstrated the potential of the current study design for accurate genomic predictions. For harvest date, the currently reported predictive ability of 0.78 was only slightly higher than the accuracy of 0.75 obtained with the initial apple REFPOP dataset measured during one year [36], but these accuracies showed a considerable improvement over other accuracies of approximately 0.5–0.6 reported elsewhere [22, 23, 29]. As shown before [36], these results underline the suitability of apple REFPOP design for the application of genomic prediction.

Opposite to harvest date and red over color, the predictive ability of yellow color and russet cover was low, although the genotypic effects explained 45% and 47% of the phenotypic variance, respectively. The across-environment clonal-mean heritability of russet cover was high (0.97), while the heritability for yellow color was slightly lower (0.81, Figure 7). Yellow color showed a moderate phenotypic clonal values correlation of 0.55 with russet cover, suggesting that the phenotyping device might have classified some russet skin as yellow color. Symptoms of powdery mildew could have been misinterpreted as russet skin. The decreased performance of genomic prediction models might stem from inaccurate phenotyping methods, insufficient SNP density in the associated regions, or other factors, all of which were outside the scope of this work.

All main-effect univariate genomic prediction models as well as other genomic prediction models compared in this study depended on predictions of clonal values obtained during the phenotypic data analysis. Further extension of the models should consider if adding a term to account for the permanent non-genetic effect of the tree over years into the mixed-effects models (Equation 1) improves accuracy of the clonal values predictions and so increases the genomic predictive ability.

### Role of genotype by environment interactions in multi-environment univariate genomic prediction

The multi-environment univariate genomic prediction models either assumed that effects of markers were the same across environments (across-environment G-BLUP, called here G-BLUP.E) or additionally estimated marker effects separately for each environment and thus considered the G × E (marker by environment interaction G-BLUP, called here G-BLUP.E.G × E) [42]. The average accuracy of the G-BLUP.E.G × E model across traits was only slightly higher than the accuracy of the G-BLUP.E. In contrast, the G-BLUP.E.G × E model had substantially greater predictive ability than the G-BLUP.E model when applied in wheat [42]. In the latter study, a productivity trait was measured under simulated conditions of mega-environments and the effect of G × E explained up to ~60% of the phenotypic variance [42]. Our work only focused on European environments and the largest proportion of phenotypic variance assigned to G × E was 24% for a productivity trait (flowering intensity). Furthermore, the average proportion of G × E across traits was approximately 12%, which may explain the mostly negligible differences between the G-BLUP.E and G-BLUP.E.G × E models. Our results were in line with the low interaction of additive genetic effects with location of up to ~6% obtained for apple fruit quality traits measured at two locations in New Zealand [33], the generally stable genetic effects found for apple fruit quality traits assessed across the commercial production region of the State of Washington [61], and the limited G × E reported for fruit maturity timing in sweet cherry across continents [62]. For approximately half of the tested traits, the G-BLUP.E.G × E did not outperform G-BLUP.E. For these traits, the G-BLUP.E ignoring G × E may be sufficient to account for the environmental effects across European sites because it is computationally simpler and therefore less demanding in terms of computational resources. Traits such as flowering intensity, soluble solids content, trunk increment or traits related to fruit size and russet showed an improved performance under G-BLUP.E.G × E when compared to G-BLUP.E. For traits where the predictive ability was greater for the G-BLUP.E.G × E model compared to the G-BLUP.E model, the G × E should be considered in marker effects estimated separately for each environment when making predictions across environments. The highest improvement of predictive ability with G-BLUP.E.G × E when compared to G-BLUP.E was found for flowering intensity, the difference between the models amounting to 0.07 (Figure 6b). This result might be explained by the highest contribution of G × E to the phenotypic variance of flowering intensity among all traits (Figure 7). A comparably high contribution of G × E was also found for weight of fruits and number of fruits, though no improvement with G-BLUP.E.G × E model was observed for these traits. When comparing the relative contributions of variance components to the phenotypic variance of flowering intensity, weight of fruits and number of fruits, the proportions of G × E were similar in the three compared traits, but the effects of genotype and environment explained a higher proportion of the variance for weight of fruits and number of fruits than for flowering intensity. This may have contributed to the surprisingly lower accuracy of the G-BLUP.E.G × E model when compared with G-BLUP.E for weight of fruits and number of fruits, but additional investigations may be needed to clarify this result in the future.

The G-BLUP.E.G × E model assumes positive correlations between environments and is therefore mostly suitable for the joint analysis of correlated environments [42, 63]. As shown by Lopez-Cruz *et al*. [42] and in our study, this assumption of G-BLUP.E.G × E resulted in the best model performance for traits showing high positive correlations of the adjusted phenotypic values of each genotype between environments (here harvest date and red over color) and the worst performance for traits exhibiting low correlations of the adjusted phenotypic values of each genotype between environments (here flowering intensity and trunk increment, Figure 6b, Supplementary Table 2, Supplementary Figure 3). For flowering intensity and trunk increment, multivariate prediction of the environments or prediction with a different G × E model not assuming positive correlations between environments might be more appropriate than the currently applied approach [42, 64].

### Multivariate models as a useful element in the genomic prediction toolbox

Multivariate (also called multi-trait) models were shown to improve predictive ability for traits that are costly to phenotype when a correlated trait less expensive to phenotype was available [45]. In our study, when the predictive ability of the main-effect multivariate model MTM.UN was compared with the baseline model G-BLUP, several combinations of related and unrelated traits led to increased accuracy. For the related traits with a high genomic breeding values correlation (Figure 2a), prediction of traits measured at one location were often improved when a related trait measured across different locations was included. This was the case for the combination of floral emergence with full flowering and end of flowering and for single fruit weight combined with fruit diameter, fruit length, maximum fruit size and fruit volume. Inclusion of soluble solids content in MTM.UN resulted in increased predictive ability for russet cover, although the traits showed only a moderate genomic breeding values correlation and no obvious explanation for this result could be found. Our study supports the potential of multivariate models to borrow information that correlated traits provide about one another and identified trait combinations that can be successful under the multivariate setup.

In place of the correlated traits, environments of a single trait can be implemented in a multivariate model [46]. Compared to the multi-environment univariate genomic prediction models G-BLUP.E and G-BLUP.E.G × E, the multi-environment multivariate genomic prediction model (MTM.FA) showed the potential to perform equally well for six (CV1) and three traits (CV2) and was able to outperform both models for two (CV1) and five traits (CV2). Except for the noticeable increase in predictive ability for trunk increment under CV2 that could not be explained by our analyses, the performance of MTM.FA was similar to G-BLUP.E and G-BLUP.E.G × E, which establishes the multivariate model as a useful tool for multi-environment genomic prediction in apple.

### Two approaches to genomic prediction addressed with cross-validation scenarios

The cross-validation scenarios CV1 and CV2 were applied with multi-environment genomic prediction models to test two genomic prediction approaches typically faced in breeding. The CV1 imitated evaluation of breeding material that was yet untested in field trials. The CV2 was implemented to simulate incomplete field trials where breeding material was evaluated in some but not all target environments. More specifically, the CV2 investigated a situation where the breeding material has been evaluated at one location (the breeding site, in this case Switzerland) and the material’s potential over other European sites was predicted without its assessment in a multi-environment trial, which may increase selection efficiency at latter stages of evaluation. As CV2 provided more phenotypic information to the models than CV1, a higher genomic predictive ability was found under CV2 when compared with CV1, which was anticipated [33, 42]. The CV2 was tested by calibrating the model with Swiss observations only. The application of CV2 could be extended to other apple REFPOP locations to provide useful information for the breeding programs located at these sites. The choice of cross-validation scenario did not affect the general ranking of the average genomic predictive abilities estimated for the evaluated traits.

### Implications for apple breeding

Phenotypic variance decomposition into genetic, environmental, G × E and residual effects was compared with the results of GWAS and genomic prediction as well as heritability estimates. The comprehensive comparison indicated three classes of traits with contrasting genetic architecture and prediction performance. Characteristics of these trait classes and proposals for their efficient prediction strategies are described in the following paragraphs.

The first class included harvest date and red over color that showed a few loci of large effect and some additional loci of low effect, the highest predictive abilities, and the highest across-environment clonal-mean heritability among all traits. Both traits showed a very high proportion of the genotypic effect explaining ~75% of the phenotypic variance. For harvest date and red over color, the marker with the largest effect explained 52% and 59% of the phenotypic variance and all marker effects in genomic prediction captured together 88% and 85% of the phenotypic variance (i.e. genomic heritability of 0.88 and 0.85), respectively. Selection for these traits exhibiting a strong genetic effect of one locus could be done using marker-assisted selection, although only a part of the variance would be explained by a single marker. Better results can be achieved using genomic prediction, as this was able to explain a substantially larger amount of the phenotypic variance. Other traits such as fruit firmness, titratable acidity, end of flowering or traits related to fruit size and water core were grouped in the same cluster as harvest date and red over color (Figure 7). These traits showed a strong genotypic effect and a comparably low effect of environment and G × E, suggesting that selection for the traits would be efficient when performed using main-effect genomic prediction models rather than multi-environment prediction.

The second class of traits was represented by floral emergence and trunk diameter displaying a high proportion of the environmental effect (~70%) and a similar ratio of variance explained by genotypic effects compared to variance explained by G × E effects (~2.5). The genomic predictive ability did not considerably deviate from the average accuracy over all traits. Several marker associations with these traits were identified using location-specific GWAS. However, in the across-location GWAS, only one association explaining a very small part of phenotypic variance (floral emergence) or no association (trunk diameter) were discovered. Consequently, such traits predominantly driven by the effect of environment can be successfully selected based on genomic prediction, but the lack of associations stable across environments limits the applicability of marker-assisted selection to this class of traits.

In the third class, the productivity traits (flowering intensity, weight of fruits and number of fruits) showed the largest proportion of variance explained by G × E (~20%), with similar amounts of variance explained by genotypic effects for weight of fruits and number of fruits, but half as much variance explained by genotypic effects for flowering intensity (Figure 7). As a consequence, only flowering intensity showed higher predictive ability with G-BLUP.E.G × E than G-BLUP.E model. As shown above, the G × E should be considered when making predictions across environments for traits responding positively to the G-BLUP.E.G × E model, but G-BLUP.E may be sufficient for other traits to account for the environmental effects. To our knowledge, this is the first report of genomic prediction for apple yield components and our results can aid the establishment of productivity predictions in apple breeding. Other traits falling within the same cluster as the productivity traits, namely full flowering, ground color, yellow color, soluble solids content, trunk increment, and traits related to bitter pit and russet, showed a pronounced effect of environment and/or G × E (Figure 7). For the majority of these and other traits in our study, the effect of G × E was estimated based on environments that represented combinations of locations and years. However, for the part of traits measured at one location, the combinations of locations and years were effectively equal to years only. Although measurements from additional locations would improve estimation of the effect of G × E in traits with unavailable multi-location data, our results were generally able to support that multi-environment genomic prediction models can be efficient when applying genomic selection to various traits in apple. Decomposition of the effects of environment and G × E into parts associated with locations, years and their interaction could indicate whether defining breeding zones would be useful for apple, but it was out of the scope of this study.

The decision to apply either marker-assisted or genomic selection can be based on genetic architecture of traits of interest and resources available in a breeding program. For breeding of yet genetically unexplored traits, variance decomposition of historical phenotypic data prior to genomic analyses may help describe trait architecture, assign traits to one of the three classes described in the previous paragraphs, and finally determine the most appropriate method of genomics-assisted breeding. From all traits explored in this study, the marker-trait associations with large and stable effects across environments found for harvest date, flowering intensity, green color, red over color, titratable acidity, fruit firmness and trunk increment could be implemented into DNA tests for marker-assisted selection. These tests would allow for a reduction of labor costs in a breeding program when removing inferior seedlings without phenotyping [7]. Although generally requiring more statistical competences than marker-assisted selection, genomic selection can make use of both large- and small-effect associations between markers and traits when accommodating thousands of marker effects in a single genomic prediction model. For all studied traits, our results showed that marker effects estimated in genomic prediction were able to capture a larger proportion of the phenotypic variance than individual markers associated with the traits. Therefore, genomic selection should become the preferred method of genomics-assisted breeding for all quantitative traits explored in this study to ultimately increase their breeding efficiency and genetic gain.

## Conclusion

This study laid the groundwork for marker-assisted and genomic selection across European environments for 30 quantitative apple traits. The apple REFPOP experimental design facilitated identification of a multitude of novel and known marker-trait associations. Our multi-environment trial provided accurate genomics-estimated breeding values for apple genotypes under various environmental conditions. Limited G × E detected in this work suggested consistent performance of genotypes across different European environments for most studied traits. Utilizing our dataset, genomic selection of traits related to yield may lead to higher productivity and increased genetic gain in the future [37]. The genomic prediction models developed here can be readily used for selecting germplasm in breeding programs, thus providing breeders with tools increasing selection efficiency. Application of our study design to other horticultural crops such as peach [65] can promote broader use of genomics-assisted breeding in the future.

## Methods

### Plant material

Plant material in this study was comprised of the apple REFPOP, which was designed and established by the collaborators of the FruitBreedomics project [66] as described by Jung *et al*. [36]. The apple REFPOP consisted of 534 genotypes from two groups of diploid germplasm. The accession group consisted of 269 accessions of European and non-European origin representing the diversity in cultivated apple. The progeny group of 265 genotypes stemmed from 27 parental combinations produced in the current European breeding programs. In 2016, the apple REFPOP was planted in six locations representing several biogeographical regions in Europe, in (i) Rillaar, Belgium, (ii) Angers, France, (iii) Laimburg, Italy, (iv) Skierniewice, Poland, (v) Lleida, Spain and (vi) Wädenswil, Switzerland (one location per country). Every genotype was replicated at least twice per location. All plants included in this study were treated with agricultural practice common to each location. Calcium spraying was avoided due to its influence on bitter pit. Flowers were not thinned, but the fruits were hand-thinned after the June fruit drop and up to two apples per fruit cluster were retained.

### Genotyping

The plant material was accompanied by a high-density genome-wide SNP marker dataset, which was produced as reported by Jung *et al*. [36]. Briefly, SNPs from two overlapping SNP arrays of different resolution, (i) the Illumina Infinium® 20 K SNP genotyping array [20] and (ii) the Affymetrix Axiom® Apple 480 K SNP genotyping array [21], were curated and then joined applying imputation with Beagle 4.0 [67] using the recently inferred pedigrees [4]. Non-polymorphic markers were removed to obtain a set of 303 148 biallelic SNPs. Positions of SNPs were based on the apple reference genome obtained from the doubled haploid GDDH13 (v1.1) [16].

### Phenotyping

Thirty phenotypic traits related to phenology, productivity, fruit size, outer fruit, inner fruit, and vigor were evaluated at up to six locations of the apple REFPOP during up to three seasons (2018–2020). Trunk diameter was measured in 2017 in some locations, enabling for a trunk increment calculation for 2018. The traits were recorded as described in the Supplementary Methods, the measurements being performed either visually or using automatic devices (sorting machine Greefa iQS4 v.1.0, the instrument Pimprenelle (Setop, France)). Two phenology traits measured in 2018, i.e. floral emergence and harvest date, were previously analyzed by Jung *et al*. [36].

### Phenotypic data analyses

Spatial heterogeneity was modeled separately for each trait and environment (combined factor of location and year) using the spatial analysis of field trials with splines (SpATS) to correct for the replicate effects and differences due to soil characteristics [68]. Phenotypic values of traits adjusted for spatial heterogeneity within each environment were estimated at the level of trees (adjusted phenotypic values of each tree) and genotypes (adjusted phenotypic values of each genotype) as described before [36].

Further analyses were performed to estimate trait heritability and remove the effects of location and year from the collected phenotypes. The general statistical model for the following phenotypic data analyses fitted via restricted maximum likelihood (R package lme4 [69]) was:

(Equation 1) \begin{equation*} \boldsymbol{y}=\boldsymbol{X}\boldsymbol{\beta } +\boldsymbol{Zb}+\boldsymbol{\varepsilon} \end{equation*}

where $\boldsymbol{y}$ was a vector of the response variable, $\boldsymbol{X}$ the design matrix for the fixed effects, $\boldsymbol{\beta}$ the vector of fixed effects, $\boldsymbol{Z}$ the design matrix for the random effects, $\boldsymbol{b}$ the vector of random effects and $\boldsymbol{\varepsilon}$ the vector of random errors. The $\boldsymbol{b}$ was a $q\times 1$ vector assuming $\boldsymbol{b}\sim N\Big(0,\boldsymbol{\Sigma} \Big)$ where $\boldsymbol{\Sigma}$ was a variance–covariance matrix of the random effects. The assumptions for the $N\times 1$ vector of random errors were $\boldsymbol{\varepsilon} \sim N\Big(0,\boldsymbol{I}{\sigma}_{\varepsilon}^2\Big)$ with $N\times N$ identity matrix $\boldsymbol{I}$ and the variance ${\sigma}_{\varepsilon}^2$, the $N$ being the number of trees.

To assess the reliability of environment-specific data, a random-effects model was first fitted separately for each trait and environment to estimate an environment-specific clonal mean heritability. Applying the Equation 1, the response $\boldsymbol{y}$ was a vector of the adjusted phenotypic values of each tree. On the place of $\boldsymbol{X}$, a vector of ones was used to model the intercept $\beta$. The design matrix $\boldsymbol{Z}$ reflected that the genotype was the grouping factor defining the random effects**.** The environment-specific clonal mean heritability was calculated from the variance components of the random-effects model as:

(Equation 2) \begin{equation*} {H}^2=\frac{\sigma_g^2}{\sigma_p^2} \end{equation*}

where the phenotypic variance ${\sigma}_p^2={\sigma}_g^2+{\sigma}_{\varepsilon}^2/{\overline{n}}_r$ was obtained from the genotypic variance ${\sigma}_g^2$, error variance ${\sigma}_{\varepsilon}^2$ and the mean number of genotype replications ${\overline{n}}_r$. The environment-specific clonal mean heritability was used to eliminate location-year-trait combinations with a heritability value below 0.1.

For the remaining location-year combinations, a single-trait mixed-effects model following the Equation 1 was fitted to the vector of the adjusted phenotypic values of each tree as response ($\boldsymbol{y}$) to estimate the across-environment clonal mean heritability. The effects of environments, i.e. combination of location and years, were used as fixed effects and the effects of genotypes and genotype by environment interactions as random effects. Estimated variances of the model components were used to evaluate the across-environment clonal mean heritability calculated using the Equation 2 with the phenotypic variance estimated as:

(Equation 3) \begin{equation*} {\sigma}_p^2={\sigma}_g^2+\frac{\sigma_{ge}^2}{n_e}+\frac{\sigma_{\varepsilon}^2}{n_e{\overline{n}}_r} \end{equation*}

where ${\sigma}_{ge}^2$ was the genotype by environment interaction variance and ${n}_e$ represented the number of environments. As the minimum number of genotype replicates at each location was only two, the variation among genotype replicates that stemmed from differences independent of the environmental influence may not be accurately captured. This may contribute to uncertainty in the estimation of phenotypic variance components. The mixed-effects model (Equation 1) did not account for the permanent non-genetic effect of the tree over years, which could lead to inflated values of clonal-mean heritability.

To predict across-location clonal values (and location-specific clonal values when only single location data was available), an additional mixed-effects model following the Equation 1 was fitted to the adjusted phenotypic values of each tree ($\boldsymbol{y}$) using the effects of location, year and their interaction as fixed effects and the effects of genotypes as random effects. Due to the skewness of their distributions, $\boldsymbol{y}$-values of the traits weight of fruits, number of fruits and trunk diameter were log-transformed. BLUPs ($\hat{\boldsymbol{b}}$) extracted from the model were further denoted as across-location clonal values. To estimate the location-specific clonal values, a model according to the Equation 1 was fitted with a subset of the adjusted phenotypic values of each tree from single locations ($\boldsymbol{y}$) using the effects of years as fixed effects and the effects of genotypes as random effects. The across-location clonal values with the addition of location-specific clonal values for traits measured at a single location were further denoted as the global clonal values. It should be noted that due to the different estimation of the two elements of global clonal values (the use of fixed effects of location, year and their interaction to estimate the across-location clonal values versus the fixed effect of year used to estimate the location-specific clonal values), the elements of the global clonal values are not strictly comparable.

The global clonal values were used to assess phenotypic clonal values correlation as the Pearson correlation between pairs of traits. The correlation between pairs of environments within traits was calculated as the Pearson correlation between the adjusted phenotypic values of each genotype within environments. To estimate the principal component analysis biplot [70], the global clonal values were scaled and centered and their missing values for each trait were replaced with the mean of the global clonal values of the same trait. A multivariate normal distribution was assumed for the ellipses in the biplot.

### Genome-wide association studies

As one of the currently most powerful procedures for identification of loci associated with complex traits in terms of computational speed and statistical power [71, 72], the Bayesian-information and linkage-disequilibrium iteratively nested keyway (BLINK) [72] was chosen to perform the GWAS. BLINK applies two fixed effect models and one filtering process for the choice of associated markers that are not in linkage disequilibrium with each other as covariates. The process is repeated until all markers are tested and the choice of associated markers is optimized using Bayesian information criteria [72]. BLINK implemented in the R package GAPIT 3.0 [73] was applied using an $n\times m$ matrix for a population of size $n=534$ genotypes (i.e. accessions and progeny) with $m=303\,148$ markers, with across-location clonal values (across-location GWAS) or location-specific clonal values (location-specific GWAS) as the response. BLINK was used with two principal components and the minor allele frequency threshold was set to 0.05. Marker-trait associations were identified as significant for p-values falling below a Bonferroni-corrected significance threshold ${\alpha}^{\ast }=\alpha /m$ with $\alpha =0.05$ ($-{\mathit{\log}}_{10}(p)>6.74$). The proportion of phenotypic variance explained by each significantly associated SNP was assessed with a coefficient of determination (${R}^2$). The ${R}^2$ was estimated from a linear regression model, which was fitted with a vector of SNP marker values (coded as 1, 2, 3) as predictor and either the across-location clonal values or location-specific clonal values as response. GWAS based on the across-location clonal values with the addition of location-specific clonal values, in cases where traits were measured at a single location only, was further denoted as the global GWAS. The position of the last SNP on a chromosome was used to estimate chromosome length, which was used to divide each chromosome into three equal segments, i.e. top, center and bottom. The marker-trait associations were assigned to these chromosome segments based on their positions to allow for a subsequent comparison with published associations.

Previous reports on QTL mapping and GWAS in apple were reviewed to perform an extensive comparison with our GWAS results (Supplementary Table 4). Published results for traits measured similarly to the traits studied in the present work were considered, with the traits being assembled into trait groups: harvest time (harvest date and similar), flowering time (floral emergence, full flowering, end of flowering and similar), productivity (flowering intensity, weight of fruits, number of fruits and similar), fruit size (single fruit weight, fruit diameter, fruit length, maximum fruit size, fruit volume and similar), ground color (ground color, yellow color and similar), over color (red over color, green color and similar), bitter pit (bitter pit frequency, bitter pit grade and similar), russet (russet cover, russet frequency overall, at stalk, on cheek and in the eye and similar), acidity (titratable acidity and similar), sugar (soluble solids content and similar), firmness (fruit firmness and similar), water core (water core frequency, water core grade and similar) and trunk (trunk diameter, trunk increment and similar). The positions of published associations within respective chromosomes were visually assigned to the three chromosome segments, i.e. top, center and bottom. The total number of markers used was recorded (Supplementary Table 4). Where the number of overlapping markers between the maternal and paternal linkage maps was not provided in a publication, the marker numbers for both maps were summed.

In the global GWAS results, the allele frequency was studied over generations. The ancestors of genotypes were identified making use of the apple pedigrees of Muranty *et al*. [4]. For all significant marker-trait associations from the global GWAS, frequency of the allele associated with increased phenotypic value was estimated for the progeny group and for its five ancestor generations. For comparison with the allele frequency in the progeny group, allele frequency was estimated separately for the 30 accessions of the progeny group that were included in the apple REFPOP. Additionally, mean allele frequencies and standard errors were estimated for 10-times repeated resampling of 30 genotypes from the progeny group.

For major significant marker-trait associations with ${R}^2>0.1$ reported in the global GWAS, linkage disequilibrium was estimated as squared Pearson’s correlations in a window of 3000 markers surrounding each of the associations. A smaller window size was used for associations located towards the end of a chromosome.

A mixed-effects model was used for every trait to estimate proportions of phenotypic variance explained by the model components as described in Jung *et al*. [36]. The mixed-effects model following the Equation 1 was fitted to the vector of the adjusted phenotypic values of each tree as response ($\boldsymbol{y}$) using the effects of environments as fixed effects and the random effects of each SNP significantly associated with the trait (a factor of the respective SNP values), the remaining random effects of genotypes and genotype by environment interactions. In cases where traits with no marker-trait associations were found in the global GWAS, the additional random effects of significantly associated SNPs were omitted from the model. The proportion of phenotypic variance associated with the fixed effect of environment was estimated as the variance of the vector of values predicted from the model fit when all random effects were set to zero. The proportions of phenotypic variance explained by the random effects of significantly associated SNPs and genotypes were summed to obtain the genotypic variance. The SNPs significantly associated with traits and the genotypes were treated as random effects to approximate genotypic variance that was explained by the SNPs (phenotypic variance associated with the random effects of SNPs) and that remained unexplained by the SNPs (phenotypic variance associated with the random effects of genotypes). Due to the low number of levels of the random effect terms of SNPs, uncertainty in the estimation of their variance may have been introduced.

A centered and scaled table (mean 0, standard deviation of 1) of trait by proportion of phenotype variances explained by different sources (genotypic, environmental, genotype by environment interaction, and residual effects) was constructed and hierarchical clustering following Ward [74] was applied to the distance matrix of this table. The number of clusters was estimated from a dendrogram, which was cut where the distance between splits was the largest.

### Genomic prediction

#### Main-effect genomic prediction

Four univariate and one multivariate main-effect genomic prediction models were used to evaluate predictive ability for 30 phenotypic traits. These models differed in the way of estimating the marker effects. For all models, the $n\times m$ matrix for a population of size $n=534$ genotypes with $m=303\,148$ markers was centered by subtracting the column means from their corresponding columns, then scaled by dividing the columns by their standard deviations, and further denoted as the additive genomic matrix $\boldsymbol{M}$. The models were fitted with the global clonal values predicted during the phenotypic data analysis.

The first univariate model examined was regression with random forest (RF) [75], which is a non-parametric model that may be able to capture non-additive effects in addition to additive effects. For RF, the response $\boldsymbol{y}$ was defined as a $n\times 1$ vector of the global clonal values and the columns of the matrix $\boldsymbol{M}$ were used as predictors. The number of decision trees in the RF was set to 500 and the number of variables randomly sampled as candidates at each split was (rounded down) $mtry=m/3$.

For the remaining three univariate and one multivariate main-effect genomic prediction models, the general random effects model was defined as:

(Equation 4) \begin{equation*} \boldsymbol{y}=1\mu +\boldsymbol{u}+\boldsymbol{\varepsilon} \end{equation*}

where $\boldsymbol{y}$ was a response vector of the global clonal values, $\mu$ was an intercept, $\boldsymbol{u}$ was a term used to specify random effects and $\boldsymbol{\varepsilon}$ was a vector of residuals.

The second univariate model BayesCπ is a parametric model, which estimates prior probability $\pi$ that a genetic marker has zero effect [76]. Following the Equation 4, the response $\boldsymbol{y}$ was a $n\times 1$ vector of the global clonal values for one trait, the term $\boldsymbol{u}=\sum_{k=1}^m{z}_k{a}_k$ with ${z}_k$ being an $n\times 1$ vector of the number of copies of one allele at the marker $k$ and ${a}_k$ being the additive effect of the marker $k$. The prior for ${a}_k$ depended on the variance ${\sigma}_{a_k}^2$ and the prior probability $\pi$ that a marker $k$ had zero effect, the priors of all marker effects having a common variance ${\sigma}_{a_k}^2={\sigma}_a^2$. The $\pi$ parameter was treated as an unknown with uniform [0, 1] prior. The random vector of residual effects followed a normal distribution $\boldsymbol{\varepsilon} \sim N(0,\boldsymbol{I}{\sigma}_{\varepsilon}^2)$ with $n\times n$ identity matrix $\boldsymbol{I}$ and the variance ${\sigma}_{\varepsilon}^2$.

The third examined univariate model, the semi-parametric Bayesian reproducing kernel Hilbert spaces regression (RKHS), is able to capture additive as well as non-additive effects and was implemented here using a multi-kernel approach [77]. The multi-kernel RKHS model was fitted according to the Equation 4 with the response $\boldsymbol{y}$ being a $n\times 1$ vector of the global clonal values for one trait, and $L=3$$n$-dimensional vectors of the random effects $\boldsymbol{u}$**.** The vectors $\boldsymbol{u}=\sum_{l=1}^L{\boldsymbol{u}}_{\boldsymbol{l}}$ followed a distribution $\boldsymbol{u}\sim N(0,{\boldsymbol{K}}_{\boldsymbol{l}}{\sigma}_{ul}^2)$, with ${\boldsymbol{K}}_{\boldsymbol{l}}$ being the reproducing kernel evaluated at the $l$th value of the bandwidth parameter $h=\{{h}_1,\dots, {h}_L\}=\{0.1,0.5,2.5\}$ and the variance ${\sigma}_{ul}^2$. For each random effect, the kernel matrix $\boldsymbol{K}=\{K({x}_i,{x}_{i^{\prime }})\}$ was an $n\times n$ matrix $K({x}_i,{x}_{i^{\prime }})=\exp \{-h\times {D}_{i{i}^{\prime }}\}$, where $\boldsymbol{D}=\Big\{{D}_{i{i}^{\prime }}=\frac{\sum_{k=1}^m{({x}_{ik}-{x}_{i\prime k})}^2}{m}\Big\}$ was the average squared-Euclidean distance matrix between genotypes, and ${x}_{ik}$ the element on line $i$ (genotype $i$) and column $k$ ($k$th marker) of the matrix $\boldsymbol{M}$. The residual effect assumed $\boldsymbol{\varepsilon} \sim N(0,\boldsymbol{I}{\sigma}_{\varepsilon}^2)$.

The fourth univariate model genomic-BLUP (G-BLUP) was fitted using a semi-parametric RKHS algorithm. To facilitate efficient incorporation of a large number of markers, the additive genomic relationship matrix traditionally used for genomic prediction was replaced in G-BLUP by a genomic relationship matrix [78]. The genomic relationship matrix $\boldsymbol{G}$ was computed as $\boldsymbol{G}=\boldsymbol{M}{\boldsymbol{M}}^{\prime }/m$ and used to fit the G-BLUP model following the Equation 4. The response $\boldsymbol{y}$ was a $n\times 1$ vector of the global clonal values for one trait. The $n$-dimensional vector of random effects followed $\boldsymbol{u}\sim N(0,\boldsymbol{G}{\sigma}_u^2)$ with variance ${\sigma}_u^2$ and the model residuals assuming $\boldsymbol{\varepsilon} \sim N(0,\boldsymbol{I}{\sigma}_{\varepsilon}^2)$.

The fifth model applied was a multivariate model with an unstructured covariance matrix of the random effect (here abbreviated as MTM.UN). The model was fitted for chosen pairs of traits using the Bayesian multivariate Gaussian model environment MTM (http://quantgen.github.io/MTM/vignette.html). The MTM.UN followed the Equation 4 with the response vector $\boldsymbol{y}$, which was a vector of the global clonal values for $t$ traits with the length $n\cdot t$ and $t=2$ being the number of traits used in the model. The vector of random effects with dimension $n\cdot t$ followed $\boldsymbol{u}\sim N(0,\boldsymbol{U}\otimes \boldsymbol{G})$ where $\boldsymbol{U}$ was an unstructured (within-genotype) covariance matrix of the random effects with dimension $t\times t$. Model residuals assumed $\boldsymbol{\varepsilon} \sim N(0,\boldsymbol{R}\otimes \boldsymbol{I})$ with $\boldsymbol{R}$ being a $t\times t$ (within-genotype) unstructured covariance matrix of the residual effect. To choose the pairs of traits for MTM.UN, the G-BLUP model was applied as described above using all genotypes to estimate genomic breeding values (estimated posterior means of random effects excluding the residuals), which were then used to obtain pairwise genomic breeding values correlations between traits. The pairs with the genomic breeding values correlations larger than 0.3 were retained for the MTM.UN analysis. In case a trait was included in more than one pair of traits, the result for the pair with the highest average predictive ability for this trait was reported.

With all models, a five-fold cross-validation repeated five times was performed, generating 25 estimates of predictive ability. The folds were chosen randomly without replacement to mask phenotypes of 20% of the genotypes in each run. Predictive ability was estimated as a Pearson correlation coefficient between phenotypes of the masked genotypes (global clonal values) and the predicted values for the same genotypes (genomic breeding values, i.e. the average predictions from all individual regression trees for RF and the estimated posterior means of random effects excluding the residuals for BayesCπ, RKHS, G-BLUP and MTM.UN).

BayesCπ, RKHS, G-BLUP and MTM.UN were applied with 12 000 iterations of the Gibbs sampler, a thinning of 5, and a burn-in of 2000 discarded samples. The RF model was implemented in the R package ranger [79], the models BayesCπ, RKHS and G-BLUP in the R package BGLR^80^ and the MTM.UN model in the R package MTM (http://quantgen.github.io/MTM/vignette.html).

#### Multi-environment genomic prediction

To explore the effects of genotypes, environments and their interaction in genomic prediction, the predictive ability for 30 traits was estimated using an across-environment and a marker by environment interaction univariate genomic prediction algorithms that assumed constant or changing random marker effects across environments, respectively. The random effects model for the examined univariate multi-environment models that were reported by Lopez-Cruz *et al*. [42] was:

(Equation 5) \begin{equation*} \boldsymbol{y}=\mathbf{1}\boldsymbol{\mu } +\boldsymbol{u}+\boldsymbol{\varepsilon} \end{equation*}

where the response $\boldsymbol{y}$ was a vector of the adjusted phenotypic values of each genotype of length $n\times r$ (with $r$ equal to the number of environments, the environments being represented as the combined factor of location and year), $\boldsymbol{\mu}$ was the vector with an intercept for each environment, $\boldsymbol{u}$ represented the vector of random effects of length $n\times r$ and $\boldsymbol{\varepsilon}$ was a vector of residuals. Of the two univariate models, the across-environment G-BLUP model (G-BLUP.E) assumed that marker effects were constant across environments with $\boldsymbol{u}\sim N(0,{\boldsymbol{G}}_{\mathbf{0}}{\sigma}_u^2)$ where ${\boldsymbol{G}}_{\mathbf{0}}=\boldsymbol{J}\otimes \boldsymbol{G}$, the $\boldsymbol{J}$ being an $r\times r$ matrix of ones. The model residuals assumed $\boldsymbol{\varepsilon} \sim N(0,\boldsymbol{I}{\sigma}_{\varepsilon}^2)$. Additionally to the constant effects of markers across environments as assumed in the previous model, the marker by environment interaction G-BLUP model (G-BLUP.E.G × E) allowed the marker effects to change across environments. The random effects were defined as $\boldsymbol{u}={\boldsymbol{u}}_{\mathbf{0}}+{\boldsymbol{u}}_{\mathbf{1}}$ where ${\boldsymbol{u}}_{\mathbf{0}}\sim N(0,{\boldsymbol{G}}_{\mathbf{0}}{\sigma}_{u0}^2)$ represented the random effects common to all environments and ${\boldsymbol{u}}_{\mathbf{1}}\sim N(0,{\boldsymbol{G}}_{\mathbf{1}})$ the random deviations of the effects for specific environments with:

$$ {\boldsymbol{G}}_{\mathbf{1}}=\left[\begin{array}{ccc}{\sigma}_{u1}^2\boldsymbol{G}& 0& 0\\ {}0& {\sigma}_{u2}^2\boldsymbol{G}& 0\\ {}0& 0& {\sigma}_{u3}^2\boldsymbol{G}\end{array}\right] $$

assuming $r=3$ here for easier notation. The model residuals assumed $\boldsymbol{\varepsilon} \sim N(0,\boldsymbol{I}{\sigma}_{\varepsilon}^2)$.

To test multivariate multi-environment genomic prediction for the traits under study, a multivariate multi-environment factor-analytic model (here abbreviated as MTM.FA) was fitted to the genomic and phenotypic data using the Bayesian multivariate Gaussian model environment MTM (http://quantgen.github.io/MTM/vignette.html). The traits measured at only one location during two seasons (full flowering, end of flowering, fruit volume, water core frequency and water core grade) were not modeled using MTM.FA because the analysis required at least three environments. Following the Equation 5, the vector of random effects assumed $\boldsymbol{u}\sim N(0,\boldsymbol{C}\otimes \boldsymbol{G})$ where $\boldsymbol{C}$ was an $r\times r$ genetic-by-environment covariance matrix. For the factor analysis, the $\boldsymbol{C}=\boldsymbol{BB}^{\prime }+\boldsymbol{\Psi}$ where $\boldsymbol{B}$ was a matrix of loadings (regressions of the original random effects into common factors) and $\boldsymbol{\Psi}$ was a diagonal matrix whose entries gave the variances of environment-specific factors. The loadings were estimated for all environments and the variance of the Gaussian prior assigned to the unknown loadings was set to 100. The model residuals assumed $\boldsymbol{\varepsilon} \sim N(0,\boldsymbol{R}\otimes \boldsymbol{I})$ with $\boldsymbol{R}$ being an $r\times r$ (within-genotype) unstructured covariance matrix of the residual effect and $\boldsymbol{I}$ the $n$-dimensional identity matrix.

The folds of a five-fold cross-validation were chosen randomly without replacement. The cross-validation was repeated under two scenarios. In the first cross-validation scenario (CV1), the phenotypes of 20% of the genotypes were masked across all environments. For the second cross-validation scenario (CV2), the phenotypes of 20% of the genotypes were masked across all environments except for three Swiss environments, i.e. phenotypes of all genotypes from the environments “CHE.2018”, “CHE.2019” and “CHE.2020” were used for model training. Ten traits were measured in only one location and therefore excluded from CV2 (i.e. full flowering, end of flowering, fruit diameter, fruit length, maximum fruit size, fruit volume, yellow color, green color, water core frequency and water core grade). Predictive ability was estimated as a Pearson correlation coefficient between the phenotypes of the masked genotypes (adjusted phenotypic values of each genotype in each environment) and the predicted values for these genotypes in each environment (estimated posterior means of random effects excluding the residuals). The correlations were estimated for each predicted environment separately. The models following the Equation 5 did not account for the permanent non-genetic effect of the tree over years.

All three multi-environment genomic prediction models were applied with 12 000 iterations of the Gibbs sampler, a thinning of 5 and a burn-in of 2000 discarded samples. The models G-BLUP.E and G-BLUP.E.G × E were implemented in the R package BGLR [80], the model MTM.FA in the R package MTM (http://quantgen.github.io/MTM/vignette.html).

### Genomic heritability

The BayesCπ model was applied for each trait as described before but trained with a full set of the global clonal values as response. The genomic heritability ${h}^2={V}_g/({V}_g+{V}_e)$ was estimated as the proportion of phenotypic variance explained by the markers, where ${V}_g$ and ${V}_e$ represented the amount of phenotypic variance explained and unexplained by the markers, respectively [81, 82]. The genomic heritability was calculated from the marker effects saved in each iteration of the Gibbs sampler and averaged over iterations to obtain the mean genomic heritability per trait.

## Acknowledgements

The authors thank the field technicians and staff, especially Sylvain Hanteville, at INRAe IRHS and Experimental Unit (UE Horti), Angers, France, and technical staff at other apple REFPOP sites for the maintenance of the orchards and phenotypic data collection. We thank Dr. Graham Dow for English language editing. Phenotypic data collection was partially supported by the Horizon 2020 Framework Program of the European Union under grant agreement No 817970 (project INVITE: “Innovations in plant variety testing in Europe to foster the introduction of new varieties better adapted to varying biotic and abiotic conditions and to more sustainable crop management practices”). This work was partially supported by the project RIS3CAT (COTPA-FRUIT3CAT) financed by the European Regional Development Fund through the FEDER frame of Catalonia 2014-2020 and by the CERCA Program from Generalitat de Catalunya. We acknowledge financial support from the Spanish Ministry of Economy and Competitiveness through the “Severo Ochoa Programme for Centres of Excellence in R&D” 2016-2019 (SEV-20150533) and 2020-2023 (CEX2019-000902-S). C.D. was supported by “DON CARLOS ANTONIO LOPEZ” Abroad Postgraduate Scholarship Program, BECAL-Paraguay. We dedicate this paper to Prof. Edward Zurawicz of the National Institute of Horticultural Research in Skierniewice, Poland who co-promoted this study, but sadly recently passed away.

## Author contributions

M.J.A., W.G., F.L., H.M. and A.P. conceived the research plans; B.S., H.M. and A.P. supervised the project; M.J., B.K., M. Roth, M.J.A., A.A., M.A., M.L., N.S., M. Rymenants, F.D., C.D., C.F.F. and A.K. contributed to data collection; M.J. carried out the data analysis and wrote the article in consultation with B.K., M. Roth, H.M. and A.P.; M.J.A., A.A., W.G., N.S., M. Rymenants, F.L. and B.S. provided critical feedback to the article.

## Data availability

All SNP genotypic data generated with the 480 K array used in this study have been deposited in the INRAe dataset archive (https://data.inrae.fr/) at https://doi.org/10.15454/IOPGYF. All SNP genotypic data generated using the 20 K array used in this study have been deposited in the INRAe dataset archive at https://doi.org/10.15454/1ERHGX. The raw phenotypic data are available in the INRAe dataset archive at https://doi.org/10.15454/VARJYJ.

## Competing interests

The authors declare no competing interests.

## Supplementary data

Supplementary data is available at *Horticulture Research Journal* online.

## Supplementary Material

Web_Material_uhac028
