# Correction to: Genetic architecture and genomic predictive ability of apple quantitative traits across environments

**DOI:** 10.1093/hr/uhac273

**Published:** 2022-02-19

This is a correction to: Michaela Jung, Beat Keller, Morgane Roth, Maria Jos Aranzana, Annemarie Auwerkerken, Walter Guerra, Mehdi Al-Rifaï, Mariusz Lewandowski, Nadia Sanin, Marijn Rymenants, Frdrique Didelot, Christian Dujak, Carolina Font i Forcada, Andrea Knauf, François Laurens, Bruno Studer, Hélène Muranty, Andrea Patocchi, Genetic architecture and genomic predictive ability of apple quantitative traits across environments, Horticulture Research, Volume 9, 2022, uhac028, https://doi.org/10.1093/hr/uhac028

An error resulting from an incorrect match of datasets produced using the Illumina Infinium® 20K SNP genotyping array and the Affymetrix Axiom® Apple 480K SNP genotyping array has been discovered in the imputed genomic dataset of the apple REFPOP. Using the corrected imputed genomic dataset, the average predictive abilities obtained across traits and model runs increased by 0.008 for the benchmark main-effect model G-BLUP when compared to predictive abilities obtained with the original dataset. Based on these results, the estimated effect of the error on the published genomic predictive abilities was negligible. When repeating GWAS using the corrected dataset, differences between the original and the corrected results were observed. A revised Supplementary Table 3 containing a list of significant marker-trait associations identified using GWAS that was based on the corrected genomic dataset was provided.

