# Supplementary material for: Genetic architecture and genomic predictive ability of apple quantitative traits across environments
Source: Hortic Res. 2022 Feb 19;9:uhac028. doi: 10.1093/hr/uhac028 (PMC8976694; doi:10.1093/hr/uhac028)
Supplement: Web_Material_uhac028 [file web_material_uhac028.zip › Supplementary figures.pdf]

**Genetic architecture and genomic predictive ability of apple quantitative traits across environments**

Jung, Michaela; Keller, Beat; Roth, Morgane; Aranzana, Maria José; Auwerkerken, Annemarie; Guerra, Walter; Al-Rifaï, Mehdi; Lewandowski, Mariusz; Sanin, Nadia; Rymenants, Marijn; Didelot, Frédérique; Dujak, Christian; Font i Forcada, Carolina; Knauf, Andrea; Laurens, François; Studer, Bruno; Muranty, Hélène; Patocchi, Andrea

## Supplementary figures

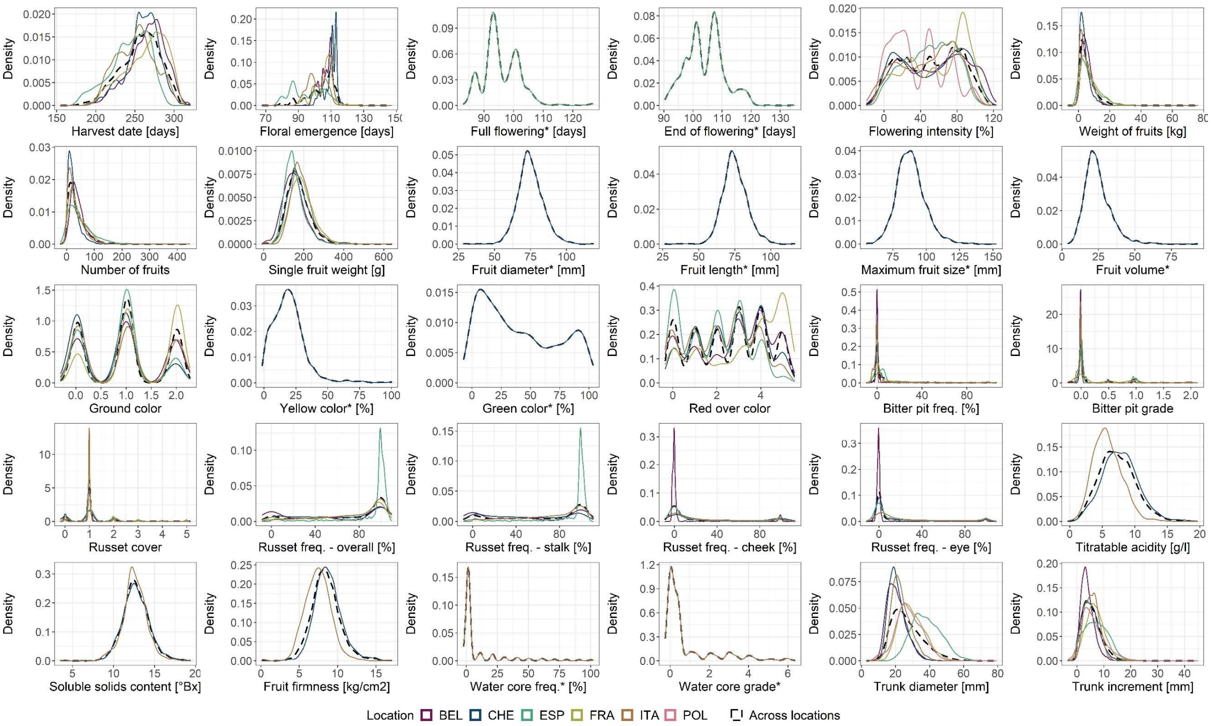


**Supplementary Figure 1:** Distributions of phenotypic values of traits adjusted for spatial heterogeneity within environments (adjusted phenotypic values of each tree), plotted per location. Traits measured at a single location are labeled with an asterisk.

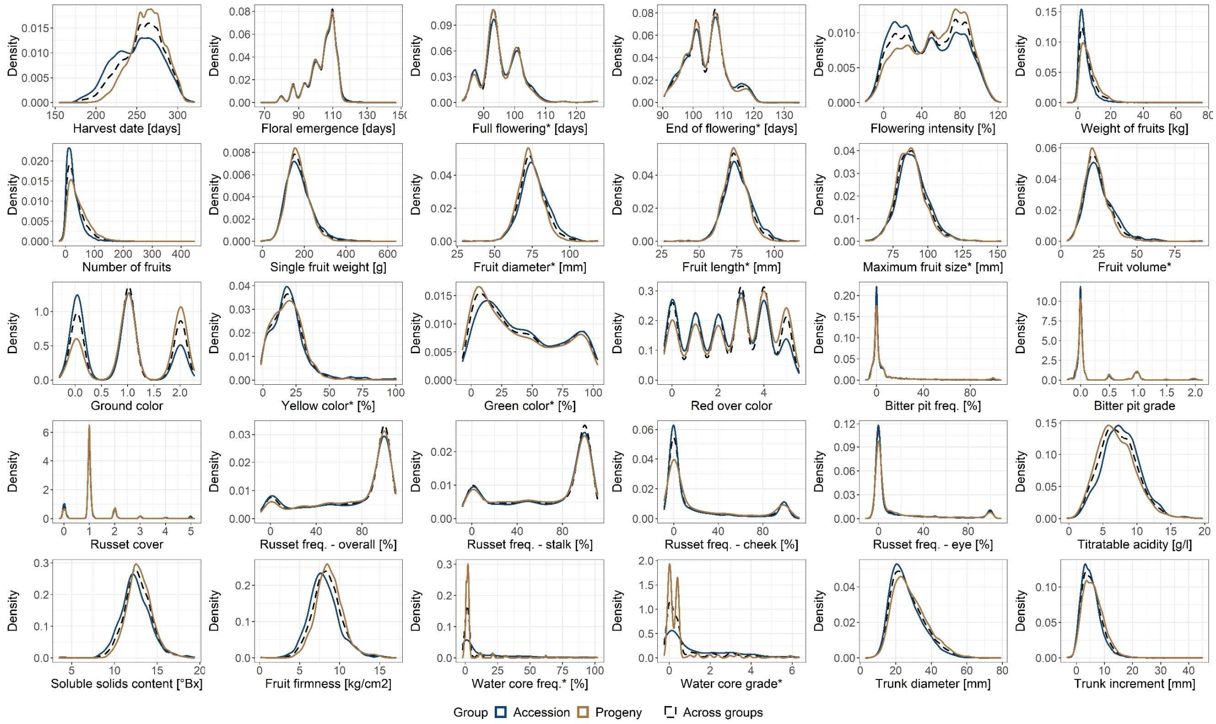


**Supplementary Figure 2:** Distributions of phenotypic values of traits adjusted for spatial heterogeneity within environments (adjusted phenotypic values of each tree), plotted per apple REFPOP group. Traits measured at a single location are labeled with an asterisk.

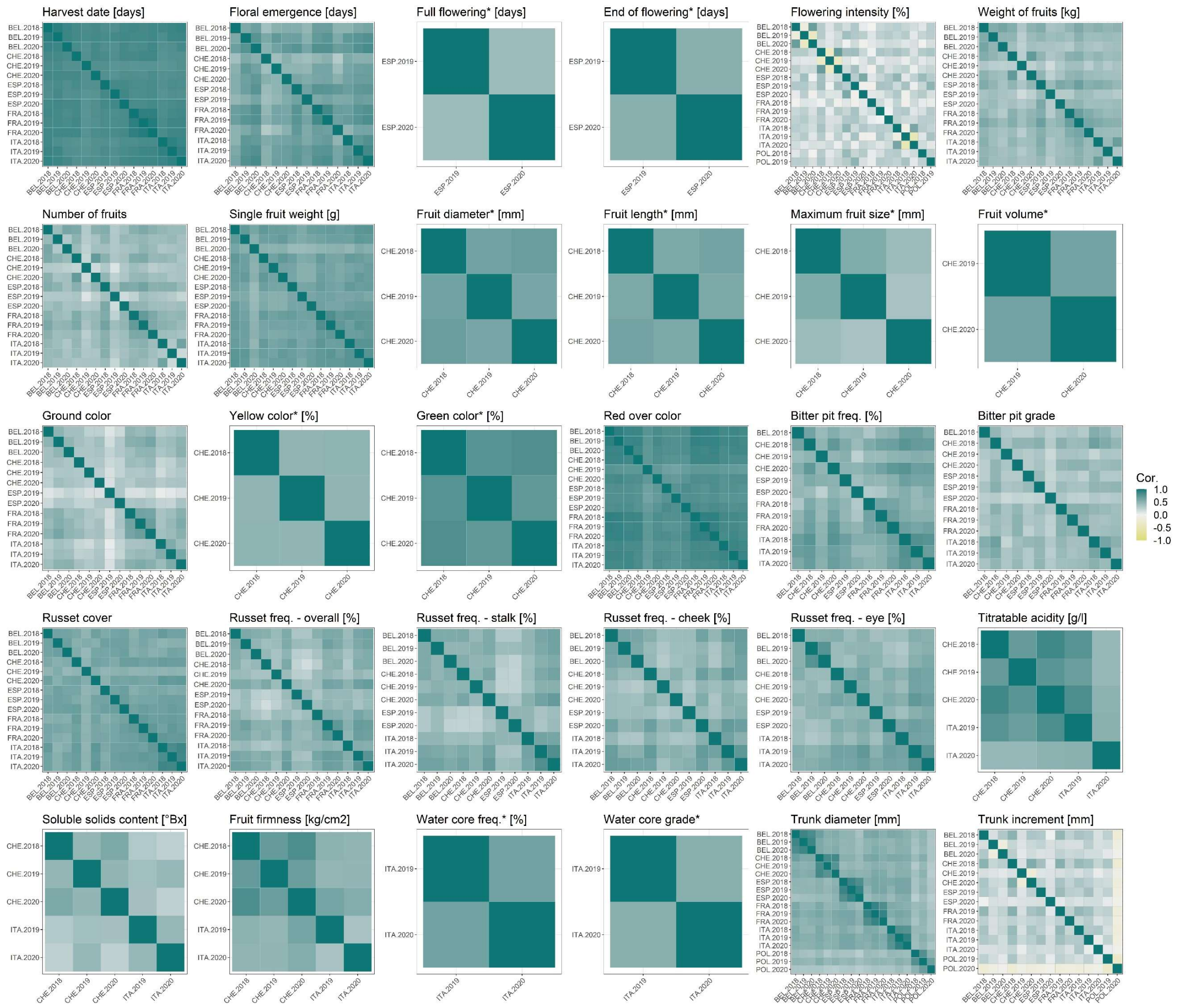


**Supplementary Figure 3:** Pairwise correlations of the adjusted phenotypic values of each genotype measured in different environments for individual traits. Traits measured at a single location are labeled with an asterisk.

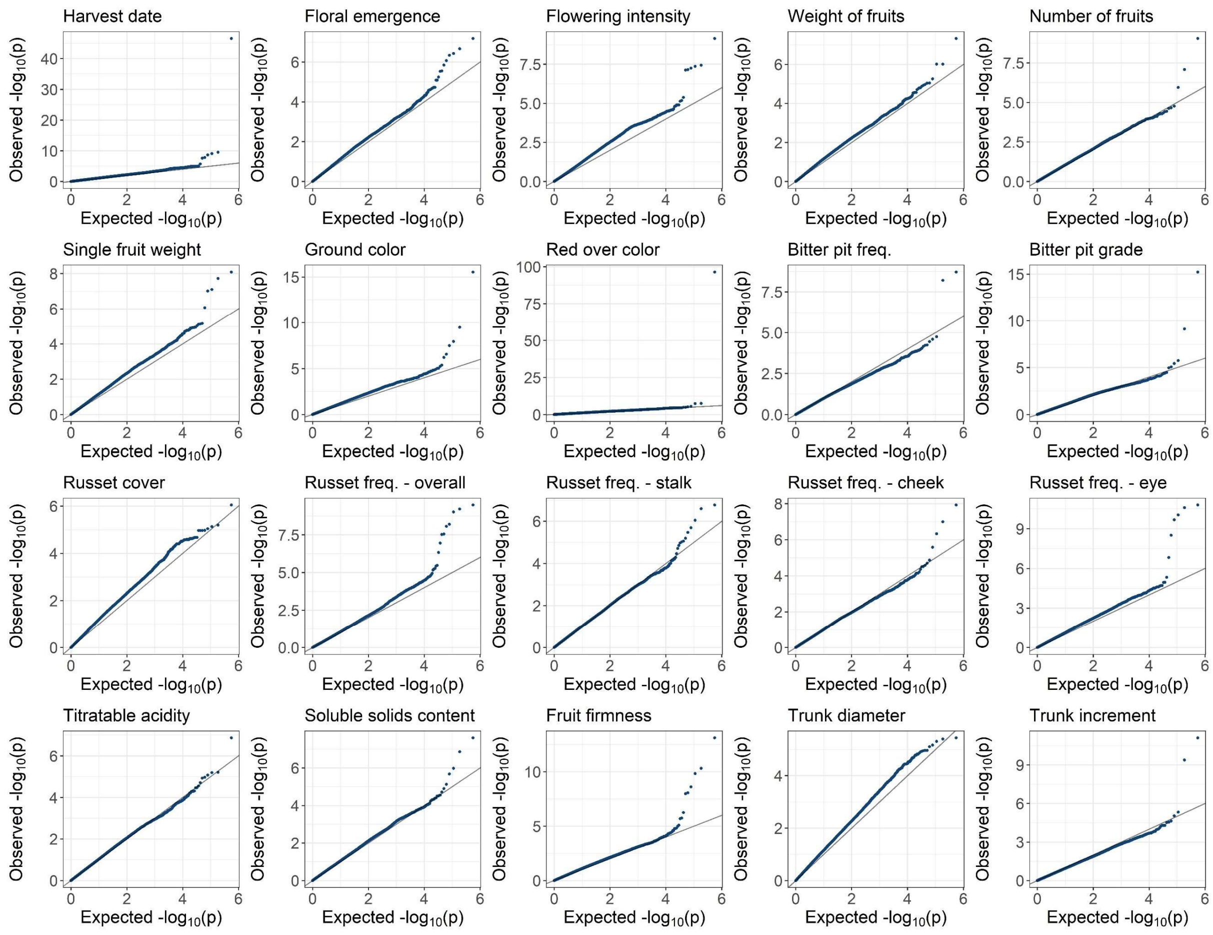


**Supplementary Figure 4:** QQ plots of the observed versus expected p-values for individual traits from the across-location GWAS.

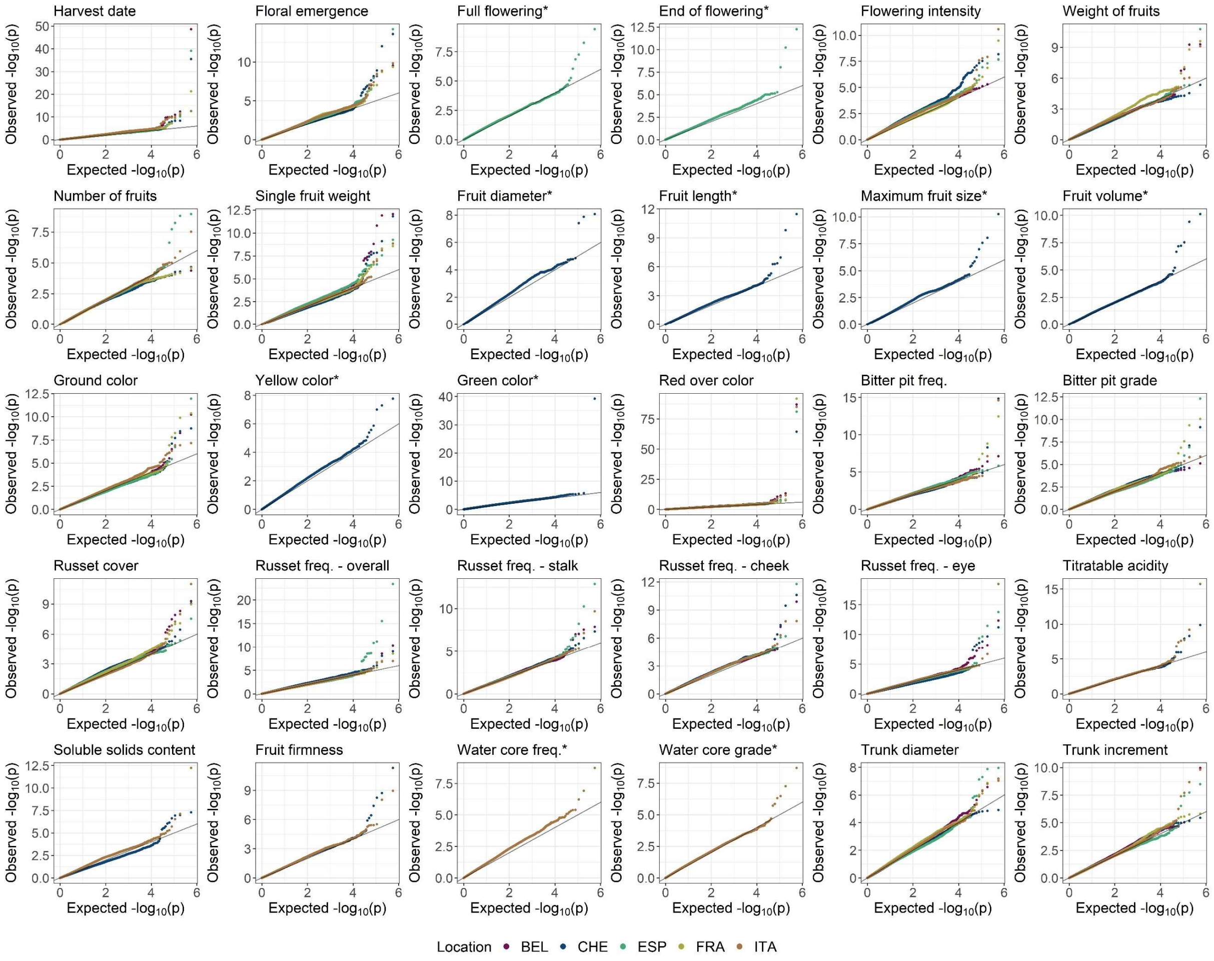


**Supplementary Figure 5:** QQ plots of the observed versus expected p-values from the location-specific GWAS for individual traits and locations. Traits measured at a single location are labeled with an asterisk.

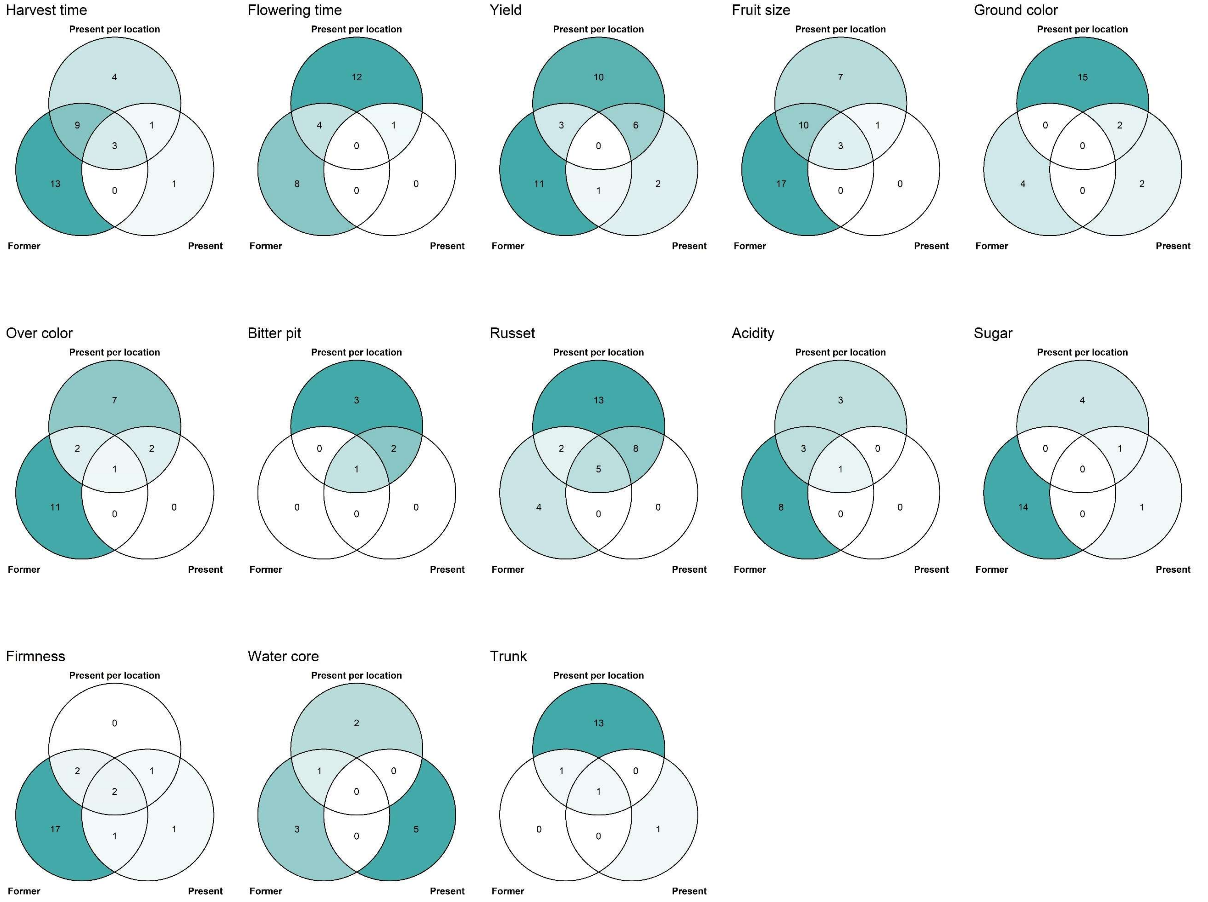


**Supplementary Figure 6:** Venn diagrams for each trait comparing the number of published associations (former, see also Supplementary Table 4) with the significant marker-trait associations from the across-locations GWAS (present, see also Supplementary Table 3) and location-specific GWAS (present (per location), see also Supplementary Table 3). The traits were assembled into trait groups based on their similarity. Color intensity reflects the number of associations per diagram area. The associations were assigned to chromosome segments (top, center, and bottom of a chromosome).

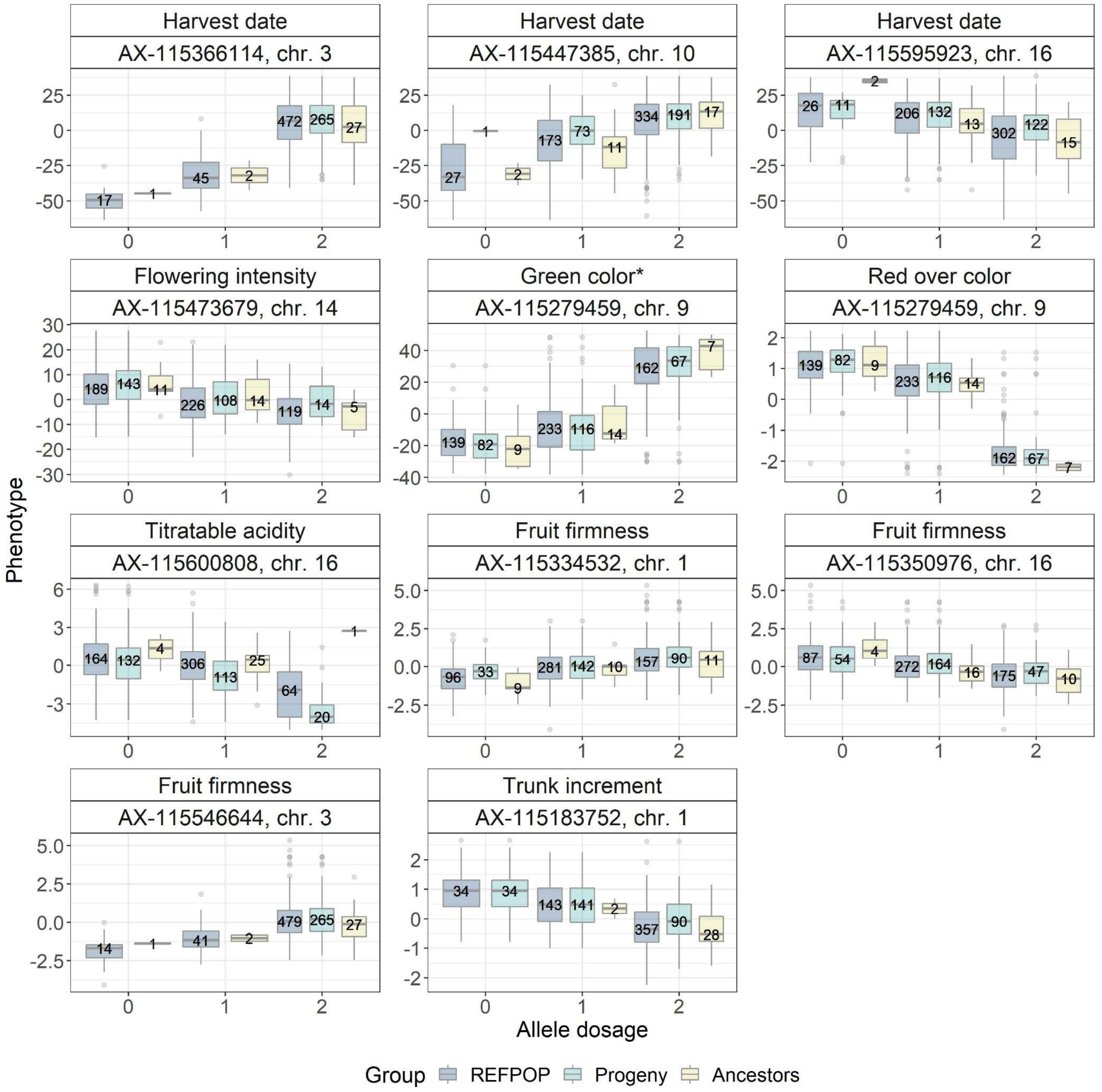


**Supplementary Figure 7:** Boxplots of phenotypes (across-location clonal values) against dosage of the reference allele (0 – reference allele, 1 – heterozygote, 2 – alternative allele) for the major significant marker-trait associations, plotted for all apple REFPOP genotypes (REFPOP), the apple REFPOP progeny group (Progeny) and 30 ancestral accessions of the progeny group included in the apple REFPOP (Ancestors). Number of genotypes of a subgroup is shown in each box. Less than nine boxplots per trait indicate that not all specific allelic combinations were present in the respective group. The trait measured at a single location is labeled with an asterisk.

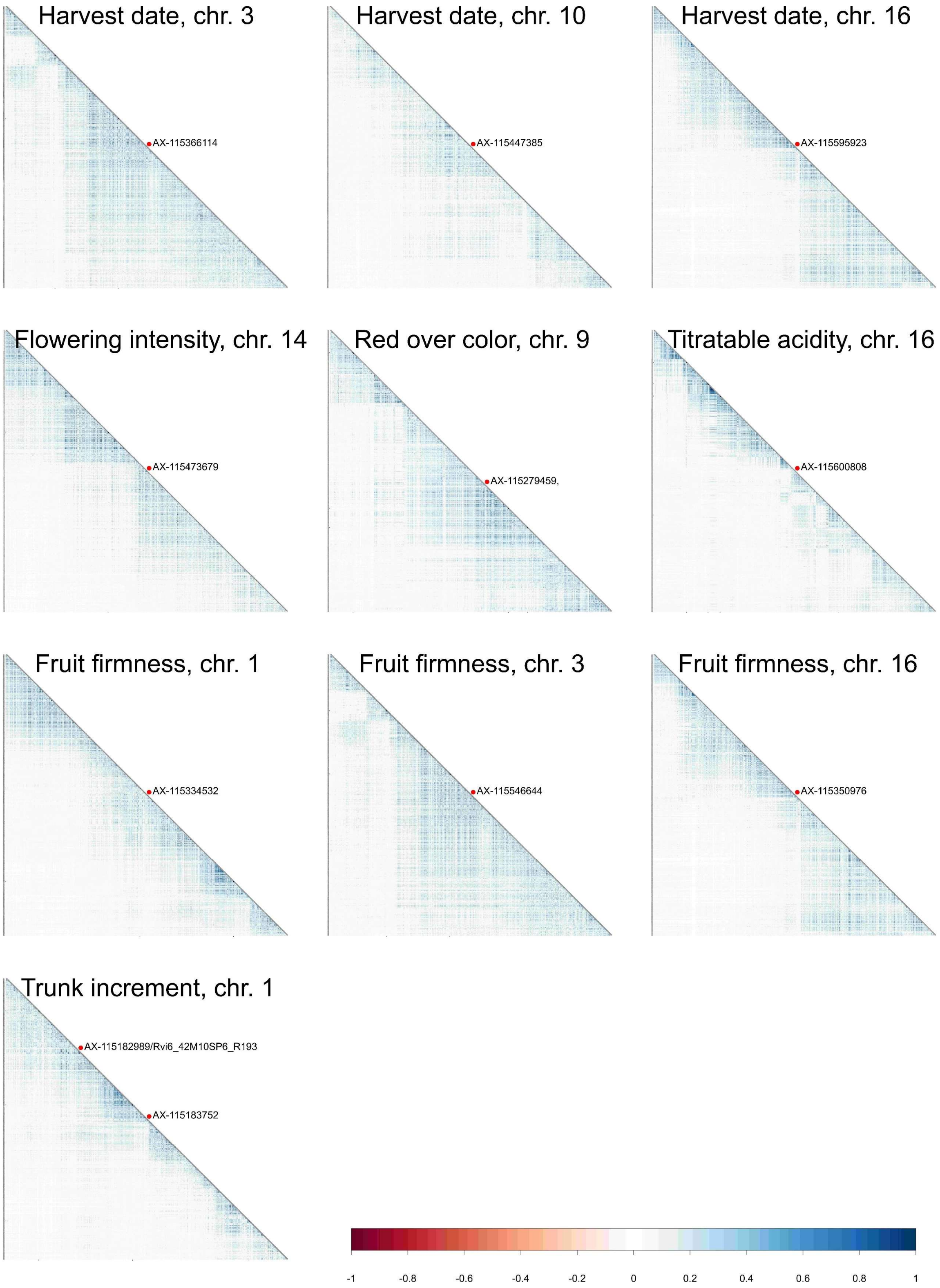


**Supplementary Figure 8:** Linkage disequilibrium estimated as squared Pearson's correlations in a window of 3,000 markers surrounding each of the major significant marker-trait associations. For the association with red over color, which corresponds to green color, only 2,736 markers were used due to the position of the association towards the end of chromosome nine. Position of a marker associated with apple scab resistance (*Rvi6*, marker AX-115182989 also known as Rvi6_42M10SP6_R193) is additionally shown in the plot of trunk increment. Physical size of the marker windows ranged between 4.1 Mb (harvest date, chromosome 10) and 5.8 Mb (trunk increment, chromosome 1). The colored scale indicates the sign and magnitude of the correlation.

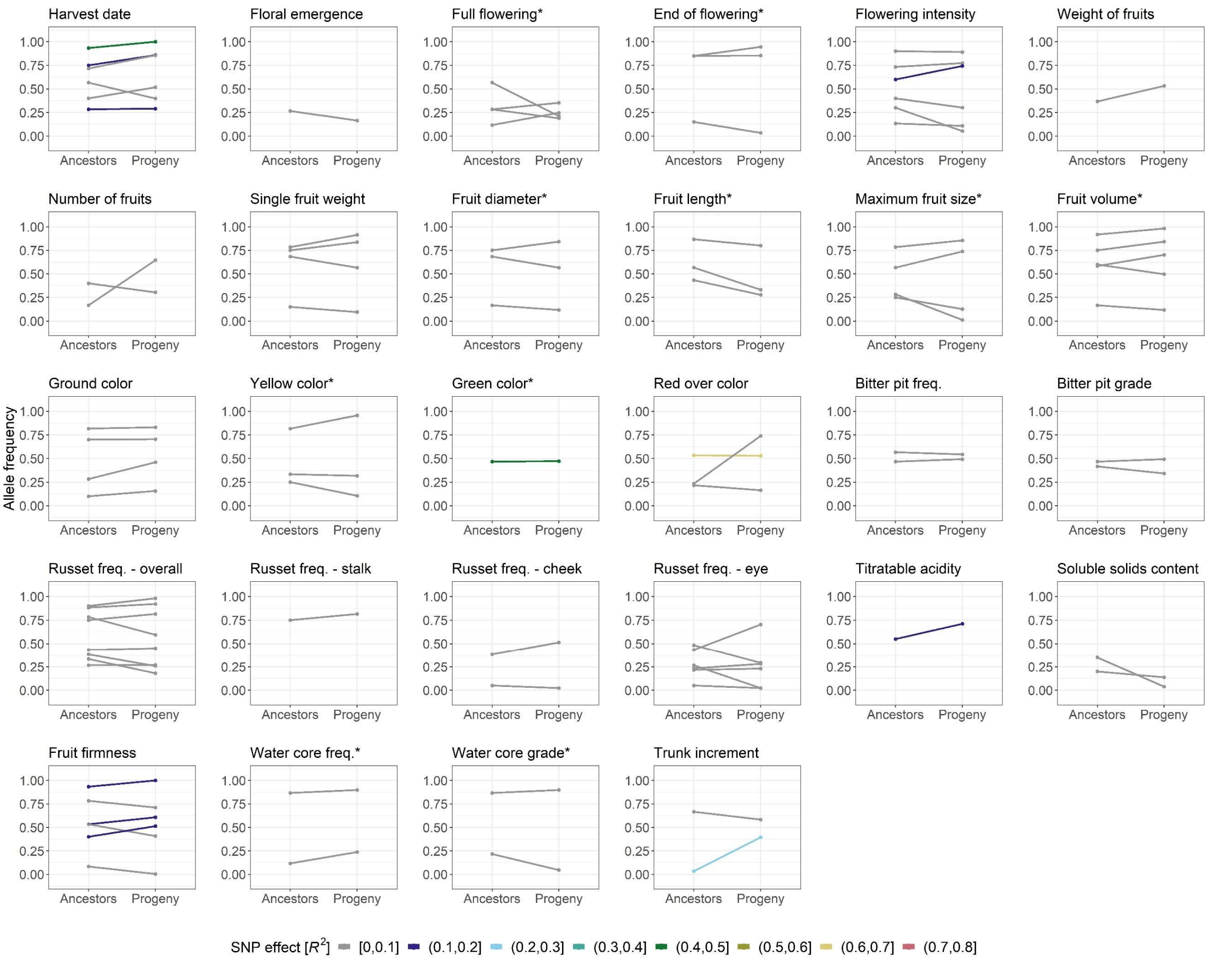


**Supplementary Figure 9:** Frequencies of alleles associated with increased phenotypic value for all significant marker-trait associations from the global GWAS. For the apple REFPOP progeny group (progeny) and its five ancestor generations (ancestors), the allele frequencies are shown as points connected with a line. Out of all known ancestors, the allele frequency was estimated for 30 accessions included in the apple REFPOP. Colors of the regression lines correspond to the part of phenotypic variance ($R^2$) explained by the associated SNPs. Traits measured at a single location are labeled with an asterisk.

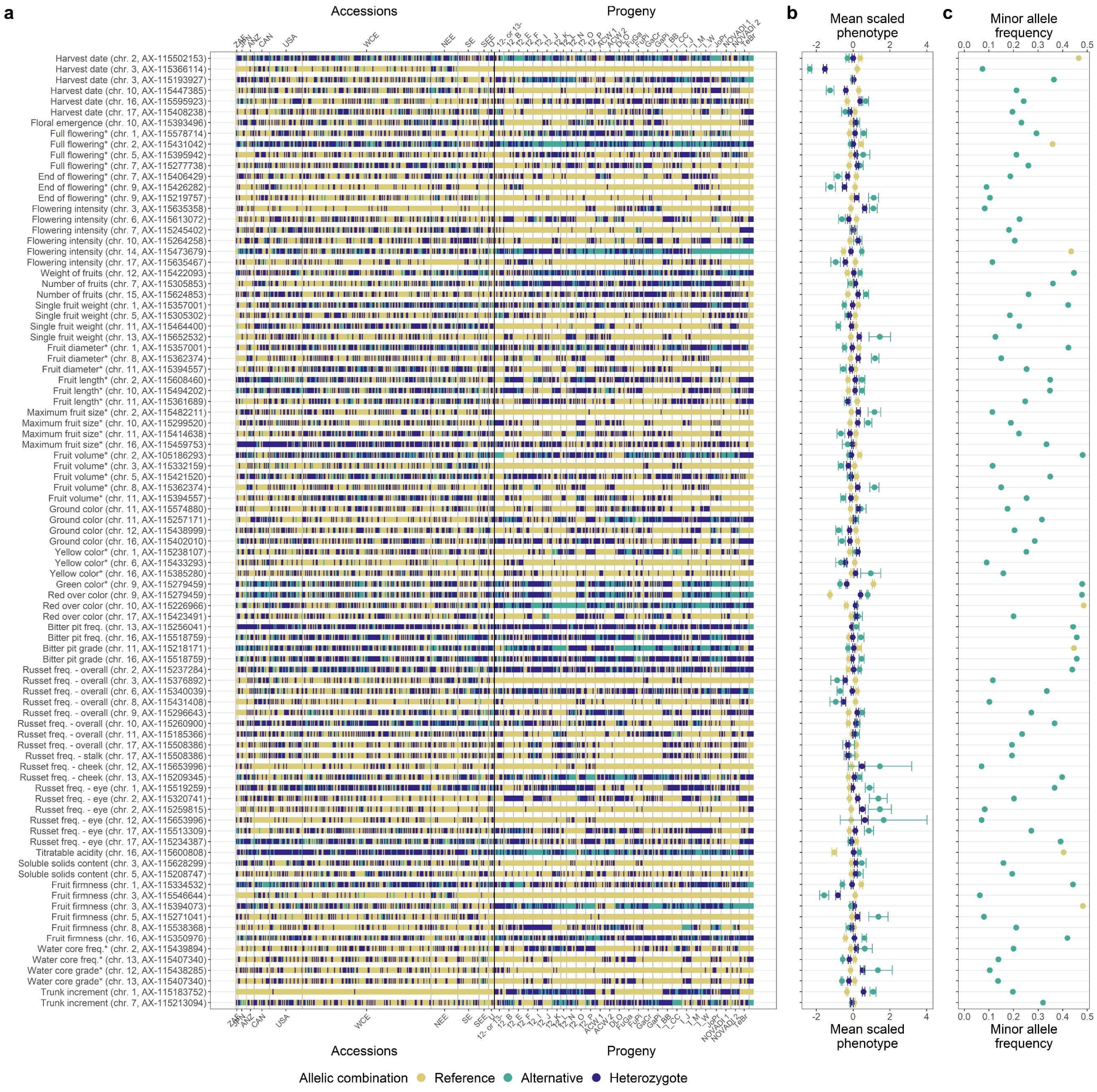


**Supplementary Figure 10: a** Allelic combinations carried by the apple REFPOP genotypes, sorted according to geographic origin of accessions (269) and affiliation of progeny (265) to parental combinations (the x-axis was labeled according to Supplementary Table 1 and 2 in Jung et al. 2020). **b** Mean scaled and centered global clonal values of traits and their standard error for each allelic combination. **c** Frequency of the minor allele in the whole apple REFPOP. **a-c** The legend and y-axis are shared between plots. In c, the color of an allelic combination corresponds to an allele of the same name. Presented are associations from the global GWAS (across-location GWAS with the addition of location-specific GWAS for traits measured at a single location only). Traits measured at a single location are labeled with an asterisk.

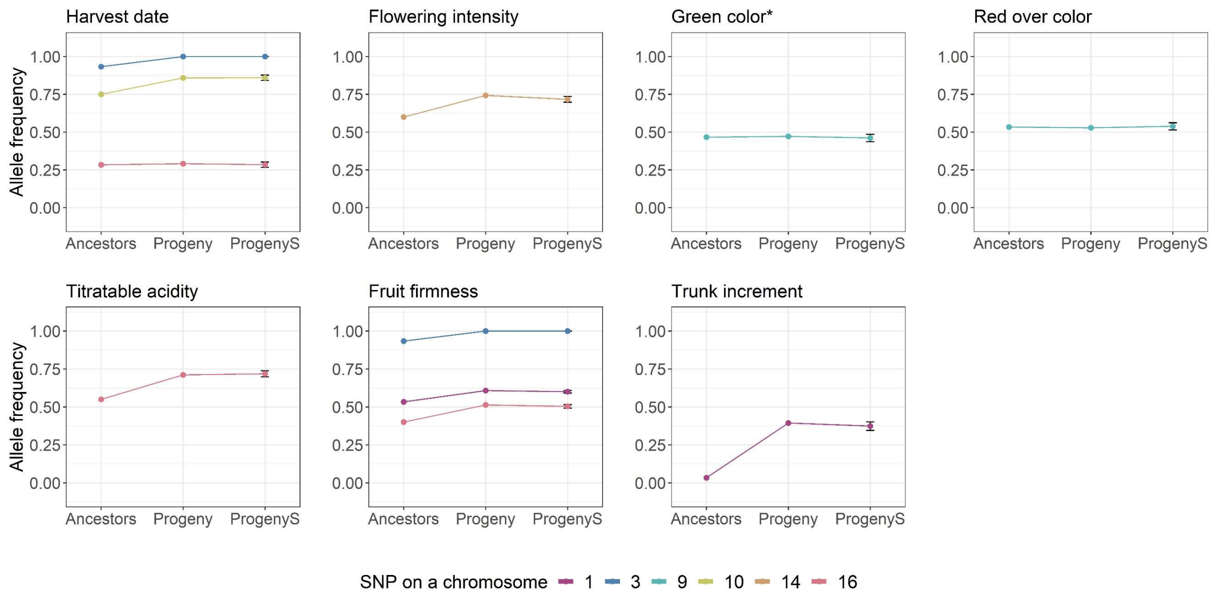


**Supplementary Figure 11**: Comparison between the results showed in Figure 5 on allele frequency dynamics of the major significant marker-trait associations ($R^2$>0.1) and the results of a resampling of 30 progeny repeated 10 times. For each association, frequency of the allele with increasing effect on trait phenotypes in the apple REFPOP is shown. The allele frequencies are shown as points connected with a line for the progeny group (Progeny), its five ancestor generations (Ancestors), and the resampled progeny group (ProgenyS). Out of all known ancestors, the allele frequency was estimated for 30 accessions included in the apple REFPOP. For ProgenyS, the mean allele frequency and standard error for the 10-times repeated resampling of 30 genotypes from the progeny group is showed. Colors of the points and lines correspond to chromosome locations of the associated SNPs. The trait measured at a single location is labeled with an asterisk.

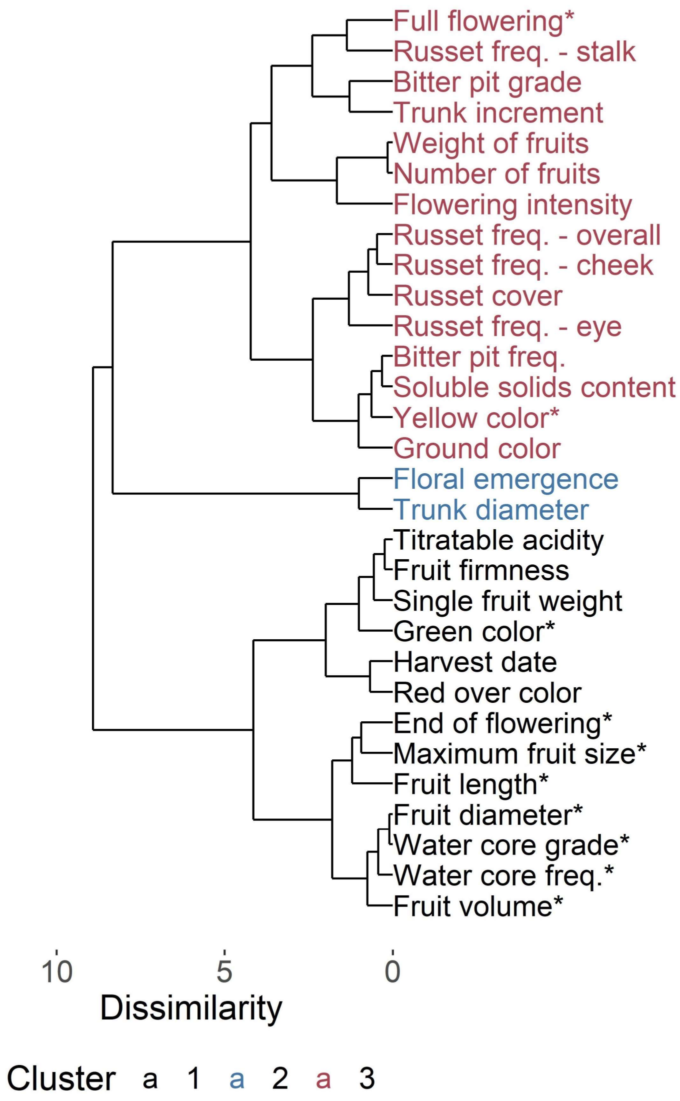


**Supplementary Figure 12:** Dendrogram based on hierarchical clustering of traits on the basis of the proportions of phenotypic variance explained by genotypic, environmental, genotype by environment interaction, and residual effects. Traits measured at a single location are labeled with an asterisk.
