# Supplementary material for: Genetic architecture and genomic predictive ability of apple quantitative traits across environments
Source: Hortic Res. 2022 Feb 19;9:uhac028. doi: 10.1093/hr/uhac028 (PMC8976694; doi:10.1093/hr/uhac028)
Supplement: Web_Material_uhac028 [file web_material_uhac028.zip › Supplementary tables - part 2.docx]

**Genetic architecture and genomic predictive ability of apple quantitative traits across environments**

Jung, Michaela; Keller, Beat; Roth, Morgane; Aranzana, Maria José; Auwerkerken, Annemarie; Guerra, Walter; Al-Rifaï, Mehdi; Lewandowski, Mariusz; Sanin, Nadia; Rymenants, Marijn; Didelot, Frédérique; Dujak, Christian; Font i Forcada, Carolina; Knauf, Andrea; Laurens, François; Studer, Bruno; Muranty, Hélène; Patocchi, Andrea

**Supplementary tables**

**Supplementary table 1:** Environments (location-year combinations) where the traits were measured (one stands for a measured environment). Locations of the measurement are labeled as: BEL – Belgium, CHE – Switzerland, ESP – Spain, FRA – France, ITA – Italy, POL – Poland. Traits measured at a single location are labeled with an asterisk.

| **Trait** | **BEL** | | | **CHE** | | | **ESP** | | | **FRA** | | | **ITA** | | | **POL** | | |
| --- | --- | --- | --- | --- | --- | --- | --- | --- | --- | --- | --- | --- | --- | --- | --- | --- | --- | --- |
|  | **2018** | **2019** | **2020** | **2018** | **2019** | **2020** | **2018** | **2019** | **2020** | **2018** | **2019** | **2020** | **2018** | **2019** | **2020** | **2018** | **2019** | **2020** |
| **Bitter pit freq.** | 1 |  |  | 1 | 1 | 1 |  | 1 | 1 | 1 | 1 | 1 | 1 | 1 | 1 |  |  |  |
| **Bitter pit grade** | 1 |  |  | 1 | 1 | 1 | 1 | 1 | 1 | 1 | 1 | 1 | 1 | 1 | 1 |  |  |  |
| **End of flowering*** |  |  |  |  |  |  |  | 1 | 1 |  |  |  |  |  |  |  |  |  |
| **Floral emergence** | 1 | 1 | 1 | 1 | 1 | 1 | 1 | 1 |  | 1 | 1 | 1 | 1 | 1 | 1 |  |  |  |
| **Flowering intensity** | 1 | 1 | 1 | 1 | 1 | 1 | 1 | 1 | 1 | 1 | 1 | 1 | 1 | 1 | 1 | 1 | 1 |  |
| **Fruit diameter*** |  |  |  | 1 | 1 | 1 |  |  |  |  |  |  |  |  |  |  |  |  |
| **Fruit firmness** |  |  |  | 1 | 1 | 1 |  |  |  |  |  |  |  | 1 | 1 |  |  |  |
| **Fruit length*** |  |  |  | 1 | 1 | 1 |  |  |  |  |  |  |  |  |  |  |  |  |
| **Fruit volume*** |  |  |  |  | 1 | 1 |  |  |  |  |  |  |  |  |  |  |  |  |
| **Full flowering*** |  |  |  |  |  |  |  | 1 | 1 |  |  |  |  |  |  |  |  |  |
| **Green color*** |  |  |  | 1 | 1 | 1 |  |  |  |  |  |  |  |  |  |  |  |  |
| **Ground color** | 1 | 1 | 1 | 1 | 1 | 1 |  | 1 | 1 | 1 | 1 | 1 | 1 | 1 | 1 |  |  |  |
| **Harvest date** | 1 | 1 | 1 | 1 | 1 | 1 | 1 | 1 | 1 | 1 | 1 | 1 | 1 | 1 | 1 |  |  |  |
| **Maximum fruit size*** |  |  |  | 1 | 1 | 1 |  |  |  |  |  |  |  |  |  |  |  |  |
| **Number of fruits** | 1 | 1 | 1 | 1 | 1 | 1 | 1 | 1 | 1 | 1 | 1 | 1 | 1 | 1 | 1 |  |  |  |
| **Red over color** | 1 | 1 | 1 | 1 | 1 | 1 | 1 | 1 | 1 | 1 | 1 | 1 | 1 | 1 | 1 |  |  |  |
| **Russet cover** | 1 | 1 | 1 | 1 | 1 | 1 | 1 | 1 | 1 | 1 | 1 | 1 | 1 | 1 | 1 |  |  |  |
| **Russet freq. - cheek** | 1 | 1 | 1 | 1 | 1 | 1 |  | 1 | 1 |  |  |  | 1 | 1 | 1 |  |  |  |
| **Russet freq. - eye** | 1 | 1 | 1 | 1 | 1 | 1 |  | 1 | 1 |  |  |  | 1 | 1 | 1 |  |  |  |
| **Russet freq. - overall** | 1 | 1 | 1 | 1 | 1 | 1 |  | 1 | 1 | 1 | 1 | 1 | 1 | 1 | 1 |  |  |  |
| **Russet freq. - stalk** | 1 | 1 | 1 | 1 | 1 | 1 |  | 1 | 1 |  |  |  | 1 | 1 | 1 |  |  |  |
| **Single fruit weight** | 1 | 1 | 1 | 1 | 1 | 1 | 1 | 1 | 1 | 1 | 1 | 1 | 1 | 1 | 1 |  |  |  |
| **Soluble solids content** |  |  |  | 1 | 1 | 1 |  |  |  |  |  |  |  | 1 | 1 |  |  |  |
| **Titratable acidity** |  |  |  | 1 | 1 | 1 |  |  |  |  |  |  |  | 1 | 1 |  |  |  |
| **Trunk diameter** | 1 | 1 | 1 | 1 | 1 | 1 | 1 | 1 | 1 | 1 | 1 | 1 | 1 | 1 | 1 | 1 | 1 | 1 |
| **Trunk increment** | 1 | 1 | 1 | 1 | 1 | 1 |  | 1 | 1 |  | 1 | 1 | 1 | 1 | 1 |  | 1 | 1 |
| **Water core freq.*** |  |  |  |  |  |  |  |  |  |  |  |  |  | 1 | 1 |  |  |  |
| **Water core grade*** |  |  |  |  |  |  |  |  |  |  |  |  |  | 1 | 1 |  |  |  |
| **Weight of fruits** | 1 | 1 | 1 | 1 | 1 | 1 | 1 | 1 | 1 | 1 | 1 | 1 | 1 | 1 | 1 |  |  |  |
| **Yellow color*** |  |  |  | 1 | 1 | 1 |  |  |  |  |  |  |  |  |  |  |  |  |

**Supplementary table 2:** Mean, minimum and maximum phenotypic correlation between environments for each trait. Traits measured at a single location are labeled with an asterisk.

| **Trait** | **Phenotypic correlation of environments** | | |
| --- | --- | --- | --- |
|  | **Mean** | **Min** | **Max** |
| **Bitter pit freq.** | 0.55 | 0.26 | 0.78 |
| **Bitter pit grade** | 0.39 | 0.19 | 0.64 |
| **End of flowering*** | 0.52 | 0.52 | 0.52 |
| **Floral emergence** | 0.62 | 0.30 | 0.84 |
| **Flowering intensity** | 0.18 | -0.49 | 0.68 |
| **Fruit diameter*** | 0.63 | 0.59 | 0.67 |
| **Fruit firmness** | 0.56 | 0.36 | 0.80 |
| **Fruit length*** | 0.59 | 0.57 | 0.62 |
| **Fruit volume*** | 0.62 | 0.62 | 0.62 |
| **Full flowering*** | 0.42 | 0.42 | 0.42 |
| **Green color*** | 0.75 | 0.71 | 0.79 |
| **Ground color** | 0.35 | 0.03 | 0.67 |
| **Harvest date** | 0.82 | 0.73 | 0.95 |
| **Maximum fruit size*** | 0.45 | 0.37 | 0.55 |
| **Number of fruits** | 0.42 | 0.10 | 0.69 |
| **Red over color** | 0.80 | 0.62 | 0.92 |
| **Russet cover** | 0.61 | 0.45 | 0.86 |
| **Russet freq. - cheek** | 0.50 | 0.30 | 0.72 |
| **Russet freq. - eye** | 0.52 | 0.32 | 0.76 |
| **Russet freq. - overall** | 0.48 | 0.15 | 0.74 |
| **Russet freq. - stalk** | 0.48 | 0.23 | 0.79 |
| **Single fruit weight** | 0.63 | 0.43 | 0.79 |
| **Soluble solids content** | 0.40 | 0.25 | 0.57 |
| **Titratable acidity** | 0.64 | 0.45 | 0.85 |
| **Trunk diameter** | 0.52 | 0.28 | 0.91 |
| **Trunk increment** | 0.16 | -0.31 | 0.55 |
| **Water core freq.*** | 0.63 | 0.63 | 0.63 |
| **Water core grade*** | 0.52 | 0.52 | 0.52 |
| **Weight of fruits** | 0.43 | 0.16 | 0.70 |
| **Yellow color*** | 0.48 | 0.47 | 0.49 |

**Supplementary table 5:** MUNQ (*Malus* UNiQue genotype code as described by Muranty et al. (2020)), original accession codes and preferred accession names for 30 ancestral accessions of the progeny group.

| **MUNQ** | **Original Code** | **Preferred Name** |
| --- | --- | --- |
| 1113 | X4194 | Red Winter |
| 1125 | 1978-136 | Lady Williams |
| 118 | 1948-236 | Reinette Clochard |
| 1236 | X2039 | O53T136 |
| 131 | 1947-143 | Reinette du Mans |
| 1478 | Priscilla_NL | Priscilla-NL |
| 163 | 1907-002 | Cox's Orange Pippin |
| 202 | 1947-086 | Franc Roseau du Valais |
| 2410 | X4355 | P7 R4A4 |
| 2422 | X6398 | TN R42A60 |
| 267 | 1972-019 | Prima |
| 29 | 1957-190 | Borowitsky |
| 30 | 1999-072 | Alexander |
| 3190 | 1921-089 | Grimes Golden |
| 32 | 1965-025 | Ingrid Marie |
| 334 | 1943-007 | Rome Beauty |
| 447 | 1974-052 | Winesap |
| 462 | 1976-149 | Wagener |
| 508 | 2006-014 | McIntosh |
| 522 | 1950-033 | Esopus Spitzenburg |
| 546 | 1953-133 | Rall's Janet |
| 548 | 1976-145 | Granny Smith |
| 57 | 1979-164 | Jonathan |
| 584 | 1971-046 | Kidd's Orange Red |
| 587 | 1974-264 | York Imperial |
| 6 | 1950-123 | Wealthy |
| 65 | 1971-054 | Golden Delicious |
| 739 | 1976-144 | Gala |
| 787 | 1963-025 | Newtown Pippin |
| 81 | 1947-050 | Prinzen Apfel |
